# Boosting near-infrared-triggered photon upconversion in optical nanomaterials *via* lanthanide-doped nanoparticle sensitization†

**DOI:** 10.1039/d5sc00937e

**Published:** 2025-04-10

**Authors:** Jiangshan Luo, Junjian Shen, Xingwen Cheng, Yan Liu, Xiulian Yin, Tianxi Hu, Guangxin Fan, Jianming Zhang, Wei Zheng, Xueyuan Chen

**Affiliations:** a Institute of Quantum and Sustainable Technology (IQST), School of Chemistry and Chemical Engineering, Jiangsu University Zhenjiang Jiangsu 212013 China chengxw@ujs.edu.cn; b State Key Laboratory of Structural Chemistry and Fujian Key Laboratory of Nanomaterials, Fujian Institute of Research on the Structure of Matter, Chinese Academy of Sciences Fuzhou Fujian 350002 China zhengwei@fjirsm.ac.cn xchen@fjirsm.ac.cn; c School of the Environment and Safety Engineering, Jiangsu University Zhenjiang Jiangsu 212013 China; d Yongkang Hardware Technician College Yongkang Zhejiang 321300 China

## Abstract

A broad spectrum of optical nanomaterials, including organic molecules, quantum dots, and metallic nanoparticles, has attracted great attention in fields such as biological imaging, data storage, solid-state lasers and solar energy conversion owing to their nonlinear optical properties facilitated by the two-photon absorption process. However, their nonlinear optical properties, particularly photon upconversion triggered using near-infrared light, are constrained by a limited multiphoton absorption cross-section, requiring a costly pulsed laser with high-density excitation. Herein, we present a straightforward and versatile strategy to enhance upconversion luminescence in various optical nanomaterials *via* sensitization with lanthanide-doped nanoparticles. This approach not only broadens the near-infrared responsivity of these luminescent nanomaterials but also introduces novel emission profiles to lanthanide-doped nanoparticles, enabling multidimensional tunability in terms of wavelength, lifetime, and polarization under low-density excitation. Concentration-dependent photoluminescence spectra and decay curves reveal a radiative energy transfer upconversion mechanism. These findings provide a general strategy for controlling photon upconversion in a wide range of luminescent nanomaterials, paving the way for innovative and versatile applications in diverse fields.

## Abstract 2

A strategy of lanthanide-doped nanoparticle sensitization was developed to enhance upconversion luminescence in diverse optical nanomaterials, achieving extended near-infrared responsivity and multidimensional spectral tunability.

## Graphical abstract

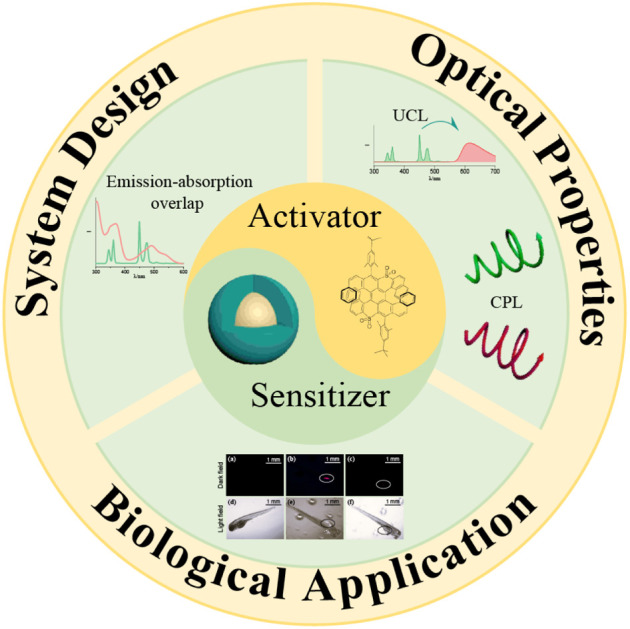

## Introduction

Photon upconversion has attracted significant attention due to its unique advantages including deeper penetration depth, higher spatial resolution, and reduced background interference.^1–5^ These characteristics make photon upconversion highly promising for applications in areas such as *in vivo* bioimaging, information security, three-dimensional displays, and solar-cell-based energy conversion.^6–12^ Currently, a broad range of optical nanomaterials such as organic molecules, quantum dots, and metallic nanoparticles are capable of generating photon upconversion luminescence (UCL) through a two-photon absorption (TPA) process.^13–16^ However, TPA suffers from inherent limitations, such as low TPA efficiency and the reliance on expensive pulsed lasers operating at high excitation densities (10^6^–10^9^ W cm^−2^). So far, it remains a challenge for these optical nanomaterials to achieve efficient photon upconversion under low-density excitation.

Lanthanide-doped upconversion nanoparticles (NPs), which can convert near-infrared (NIR) photons into ultraviolet (UV) or visible light, hold great promise for applications in theranostics, multiplex sensing, and high-security-level anti-counterfeiting owing to their exceptional optical properties such as large antenna-generated anti-Stokes shift, narrow emission bands, widely tunable lifetimes, and high photostability.^17–29^ By optimizing energy transfer pathways and designing advanced core–shell nanostructures, lanthanide-doped NPs can generate efficient UCL across a broad range of wavelengths, from UV to NIR, with lifetimes extending from microseconds to milliseconds.^30–40^ These NPs exhibit significantly higher efficiency (ranging from 10^−1^ to 10^−3^) in photon upconversion, primarily due to the sequential photon absorption and energy transfer upconversion processes between the lanthanide sensitizers and activators.^41^ This enhanced efficiency enables excitation using low-cost, continuous-wave diode lasers. Drawing inspiration from recent studies on lanthanide-doped NP-sensitized perovskite quantum dots,^42^ we propose that photon upconversion can be realized by combining various optical nanomaterials with lanthanide-doped NPs. Lanthanide-doped NPs serve as effective sensitizers for luminescent nanomaterials including organic molecules, quantum dots, and metallic nanoparticles, thereby enhancing their upconversion efficiency. In turn, the tunable emission bands of luminescent nanomaterials can help extend the emission wavelengths of lanthanide-doped NPs. Previous studies have demonstrated the potential of lanthanide-doped NPs as sensitizers, primarily focusing on perovskite quantum dots as activators, with an emphasis on tuning emission wavelengths and lifetimes.^42–44^ In this work, we aim to broaden the applicability of this strategy by expanding the range of both sensitizers and activators. This approach enables multi-wavelength excitation and emission, lifetime modulation, and the generation of circularly polarized luminescence (CPL), thereby offering a more versatile and comprehensive platform for photon upconversion.

**Scheme 1:**
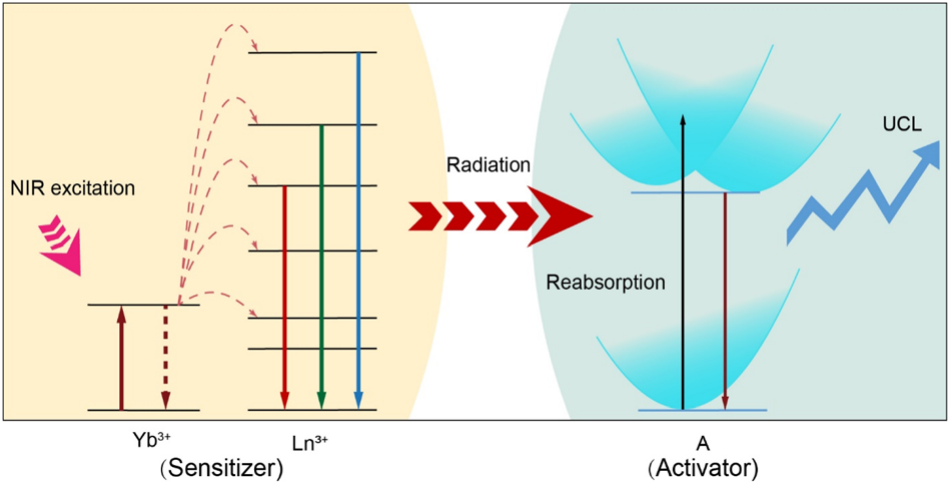
Schematic illustration of the radiative energy transfer upconversion processes in various optical nanomaterials (activator: A = chiroptical materials, transition metal complexes, lanthanide complexes, metal nanoclusters, conjugated polymers, nanoscale carbon allotropes, fluorescent dyes, and quantum dots) through lanthanide-doped nanoparticle (sensitizer) sensitization.

Herein, we present a convenient and versatile approach for fine tuning UCL in a variety of luminescent nanomaterials through sensitization using lanthanide-doped NPs. We demonstrate that the sensitization process is governed by a radiative energy transfer upconversion process. In our design (Scheme 1), we propose using lanthanide-doped NPs as general sensitizers to facilitate the photon upconversion process in a broad range of luminescent nanomaterials. The lanthanide ions (*e.g.*, Yb^3+^) absorb NIR light and are excited to a higher energy state. Through successive photon absorption and energy transfer upconversion processes, the excited Yb^3+^ ions transfer their energy to other lanthanide ions (*e.g.*, Tm^3+^ or Er^3+^). These lanthanide ions, upon absorbing the transferring energy, are excited to higher energy levels and subsequently undergo radiative transitions, emitting high-energy photons as they return to lower energy states. The emitted photons are then reabsorbed by the activators, which could be chiroptical materials, transition metal complexes, lanthanide complexes, metal nanoclusters, conjugated polymers, nanoscale carbon allotropes, fluorescent dyes, and quantum dots. These activators efficiently absorb the emitted photons and undergo further upconversion, resulting in the emission of even higher-energy photons. The integration of the sensitizers and activators not only extends the NIR responsivity of luminescent nanomaterials but also introduces novel emission profiles to lanthanide-doped NPs, with multi-dimensional tunability in parameters such as wavelength, lifetime, and polarization under low power NIR irradiation. This approach overcomes the inherent restrictions of individual luminescent nanomaterials and lanthanide-doped NPs, thereby unlocking new opportunities for materials and device engineering.

## Results and discussion

### Design of a lanthanide-doped NP-sensitized upconversion system

In the design of UCL systems, our methodology remains fundamentally governed by two critical parameters: (1) activator selection (type and concentration) and (2) sensitizer optimization for both excitation wavelength absorption and efficient energy transfer to activators. The initial design phase requires careful selection of activators based on target photophysical properties, including emission wavelength, intensity, and lifetime characteristics. The radiative energy transfer mechanism necessitates precise spectral overlap between sensitizer emissions and activator absorption bands to maximize energy transfer efficiency. This spectral matching principle constitutes the cornerstone for optimizing UCL performance. Our system architecture incorporates diverse activator candidates ranging from chiroptical materials and transition metal complexes to advanced nanomaterials, including lanthanide complexes, metal nanoclusters, conjugated polymers, nanoscale carbon allotropes, fluorescent dyes, and quantum dots. Regarding sensitizer engineering, lanthanide-doped NPs hold great promise due to their unique electronic structures, which enable tunable excitation wavelengths. For instance, Yb^3+^/Er^3+^(Tm^3+^)-doped NPs exhibit UCL emission spanning UV-vis-NIR spectral regions under 980 nm excitation. Er^3+^- and Nd^3+^-doped NPs achieve visible-NIR emissions under 1532 nm and 808 nm excitation, respectively. Our systematic analysis reveals that effective lanthanide-doped NP-based sensitizers must simultaneously satisfy two key criteria: (i) strong NIR absorption at designated excitation wavelengths and (ii) optimized energy transfer pathways to target activators.

### Lanthanide-doped NP-sensitized helicene

**Fig. 1:**
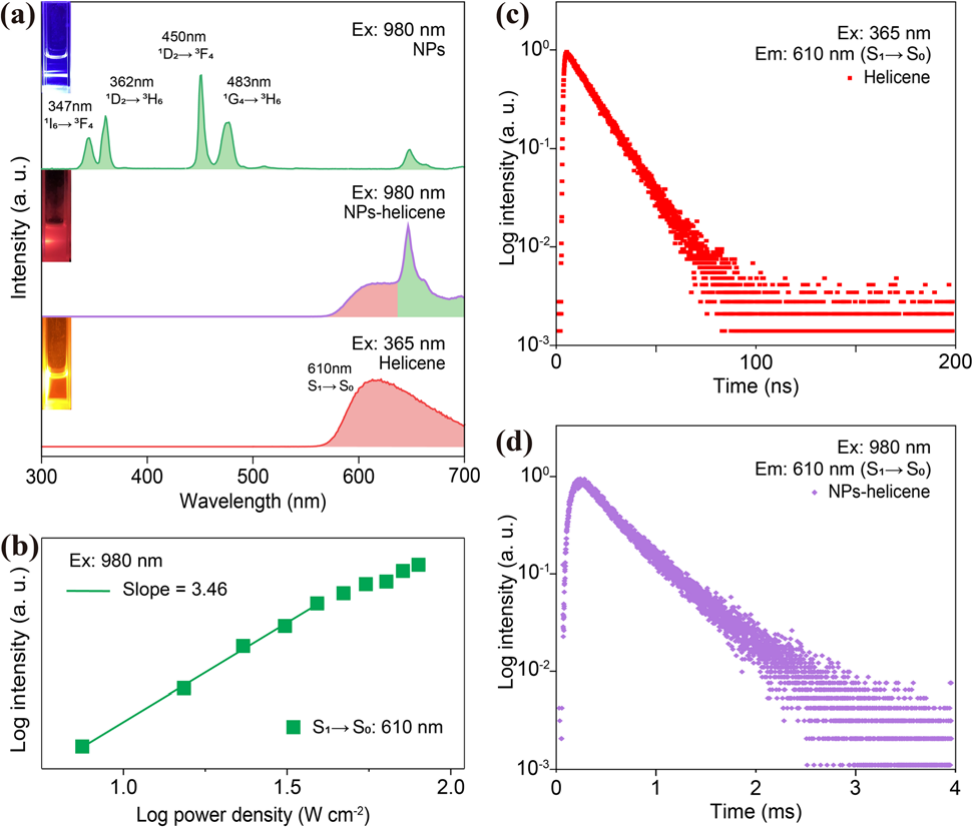
(a) PL spectrum of helicene under 365 nm excitation, and UCL spectra of the NPs (green) and NPs-helicene (purple) under 980 nm excitation at a power density of 22 W cm^−2^. (b) Power density dependence of NPs-helicene at 610 nm, indicating a four-photon population process. (c) PL decay curves of helicene at 610 nm under 365 nm excitation. (d) UCL decay curves of NPs-helicene at 610 nm under 980 nm excitation.

To investigate lanthanide-doped NP-sensitized upconversion, we synthesized core–shell NPs with the composition α-NaLuF_4_:33%Gd/49%Yb/0.5%Tm@NaLuF_4_. The morphology, elemental distribution, and structural integrity of the NPs were confirmed through transmission electron microscopy (TEM), energy-dispersive X-ray spectroscopy (EDS), and X-ray diffraction (XRD) analyses (ESI, Fig. S1 and S2†). The introduction of an outer inert shell layer resulted in a 14-fold enhancement in the UCL within the blue-violet region (ESI, Fig. S3†), accompanied by a millisecond-level luminescence lifetime (ESI, Fig. S4†). As a representative activator, we employed a sulfone-containing double [7]helicene (referred to as helicene for convenience) (ESI, Fig. S5†).^45^ The molecular structure of helicene was confirmed by ^1^H NMR spectroscopy (ESI, Fig. S6†). UV-vis absorption spectroscopy and density functional theory (DFT) calculations revealed a prominent absorption peak for helicene in the blue-violet region (ESI, Table S1, Fig. S7 and S8†). Additionally, the synthesized helicene exists as a racemic mixture containing enantiomers (ESI, Fig. S9†). Chiral separation and analysis confirmed that the helicene crystal consists of a pair of enantiomers, (*M*,*M*)-helicene and (*P*,*P*)-helicene, which adopt propeller-like structures with approximate C_2_ symmetry (ESI, Fig. S10 and S11†). The significant energy barrier between (*M*,*M*) and (*P*,*M*) configurations ensures the high stability of this helical structure (ESI, Fig. S12†). The NPs were uniformly dispersed with helicene in a dichloromethane solution, where they served as an internal UV or blue light source to illuminate helicene, harnessing the intense UCL of Tm^3+^. Fig. 1a shows UCL spectra of NPs-helicene under 980 nm laser excitation, compared with the spectra of helicene under 365 nm UV lamp excitation and individual core–shell NPs under 980 nm laser excitation. The dominant peak at approximately 610 nm in NPs-helicene corresponds to the characteristic emission of helicene. Meanwhile, the UV-blue emission from Tm^3+^ in NPs is selectively quenched due to the absorption of helicene. This phenomenon indicates effective energy transfer from NPs to helicene, thereby achieving photon upconversion through the NP-sensitized process under NIR laser excitation. Control experiments confirmed that helicene exhibited no response to 980 nm NIR light, while the NPs showed no emission under 365 nm violet light excitation (ESI, Fig. S13†). To optimize the sensitization efficiency of NPs, the concentration of NPs was varied while keeping the weight ratio with helicene constant in a dispersed solution under 980 nm laser excitation. The UCL spectra demonstrated that the emission peak of NPs-helicene at 610 nm reached its maximum intensity when 1 mg of helicene was sensitized using 40 mg of NPs. Further increases in the NP concentration resulted in only marginal enhancements in luminescence intensity, indicating that the energy transfer efficiency had reached its maximum value (ESI, Fig. S14†). We defined the energy transfer efficiency (*η*^ETE^) from NPs to helicene as the ratio of the number of photons emitted by Tm^3+^ and absorbed by helicene (*n*^x^_abs_) in the NP-sensitized helicene system to the total number of photons emitted by Tm^3+^ (*n*^Tm^_em_) in pure NPs under identical 980 nm continuous-wave diode laser excitation. Based on the UCL spectra, the energy transfer efficiency can be calculated as follows:1
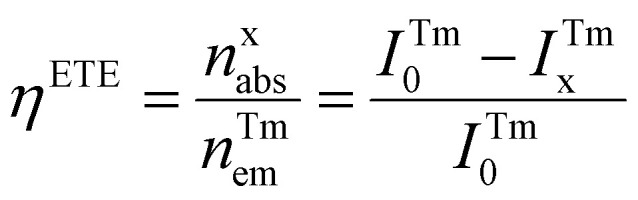
where *I*^Tm^_0_ and *I*^Tm^_x_ represent the integrated intensities of the Tm^3+^ upconversion luminescence in the absence and presence of helicene, respectively. Using this approach, we determined that the energy transfer efficiency exceeded 99.9% at a helicene-to-NP ratio of 1 : 40. The spectra of NPs and NPs-helicene under 980 nm excitation at the same power density, along with the UCL spectra of NPs-helicene under 980 nm excitation and the PL spectra of helicene under 365 nm excitation with an identical excitation power (50 mW), further confirm the high efficiency of this energy transfer process (ESI, Fig. S15†). Power-dependent measurements of NPs-helicene indicated a slope of 3.46 for the emission at 610 nm (Fig. 1b), which is attributed to the absorption of UV-blue light emissions of Tm^3+^ (^1^I_6_ → ^3^F_4_, ^1^D_2_ → ^3^H_6_, ^1^D_2_ → ^3^F_4_ and ^1^G_4_ → ^3^H_6_) by helicene (ESI, Fig. S4†). Lifetime comparisons of the characteristic emission at approximately 610 nm show that the lifetime of helicene sensitized with NPs is dramatically extended from the intrinsic nanosecond scale to milliseconds (Fig. 1c and d), with the lifetime of NPs-helicene closely matching that of the Tm^3+^ ions (ESI, Fig. S4†). It is worth noting that while the photoluminescence process of helicene remained fundamentally unchanged, its lifetime was extended due to radiative energy transfer from NPs. These experiments demonstrate the ability to manipulate the emission features of helicenes such as wavelength and lifetime, through interactions with NPs, providing valuable insights into their potential applications in photonics and optoelectronics.

**Fig. 2:**
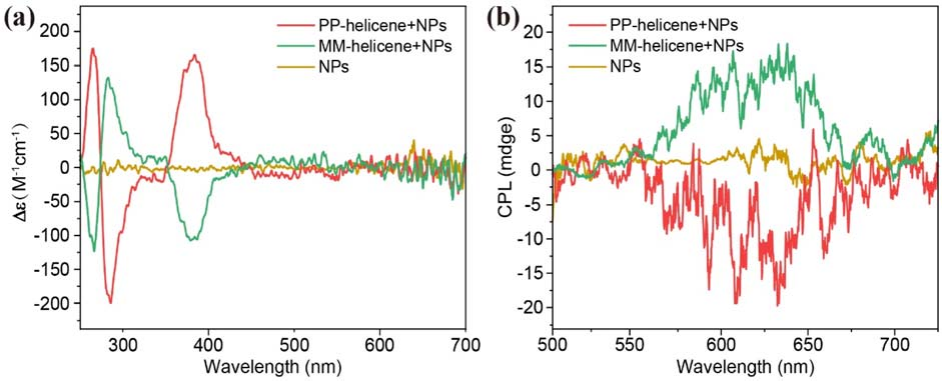
(a) Circular dichroism spectra of NPs, (*P*,*P*)-helicene + NPs, and (*M*,*M*)-helicene + NPs in dichloromethane. (b) Upconversion CPL spectra of NPs, (*P*,*P*)-helicene + NPs and (*M*,*M*)-helicene + NPs in dichloromethane (*λ*_ex_ = 980 nm).

Furthermore, NIR-triggered upconversion CPL was successfully achieved through the sensitization of lanthanide-doped NPs. As illustrated in Fig. 2a, the circular dichroism (CD) spectra of the two enantiomers (*M*,*M*)-helicene + NPs and (*P*,*P*)-helicene + NPs in dichloromethane exhibit mirror symmetry with opposite Cotton effects. A detailed comparison between the experimental and simulated CD spectra reveals that the first peak obtained by chiral separation is (*M*,*M*)-helicene + NPs, while the other peak corresponds to the (*P*,*P*)-helicene + NPs (ESI, Fig. S10†). Notably, NPs without helicene exhibit no chiroptical activity, confirming that the chirality originates from the helicene. Additionally, the upconversion CPL spectra of both (*M*,*M*)-helicene + NPs and (*P*,*P*)-helicene + NPs, when excited at 980 nm, exhibit mirror-image features within the wavelength range of 550–700 nm, further supporting the chiral nature of the emission. In contrast, the NPs alone did not exhibit any distinct CPL signals (Fig. 2b). These findings demonstrate the successful integration of helicene with lanthanide-doped NPs, enabling the modulation of CPL triggered by NIR excitation.

### Mechanistic investigation

**Fig. 3:**
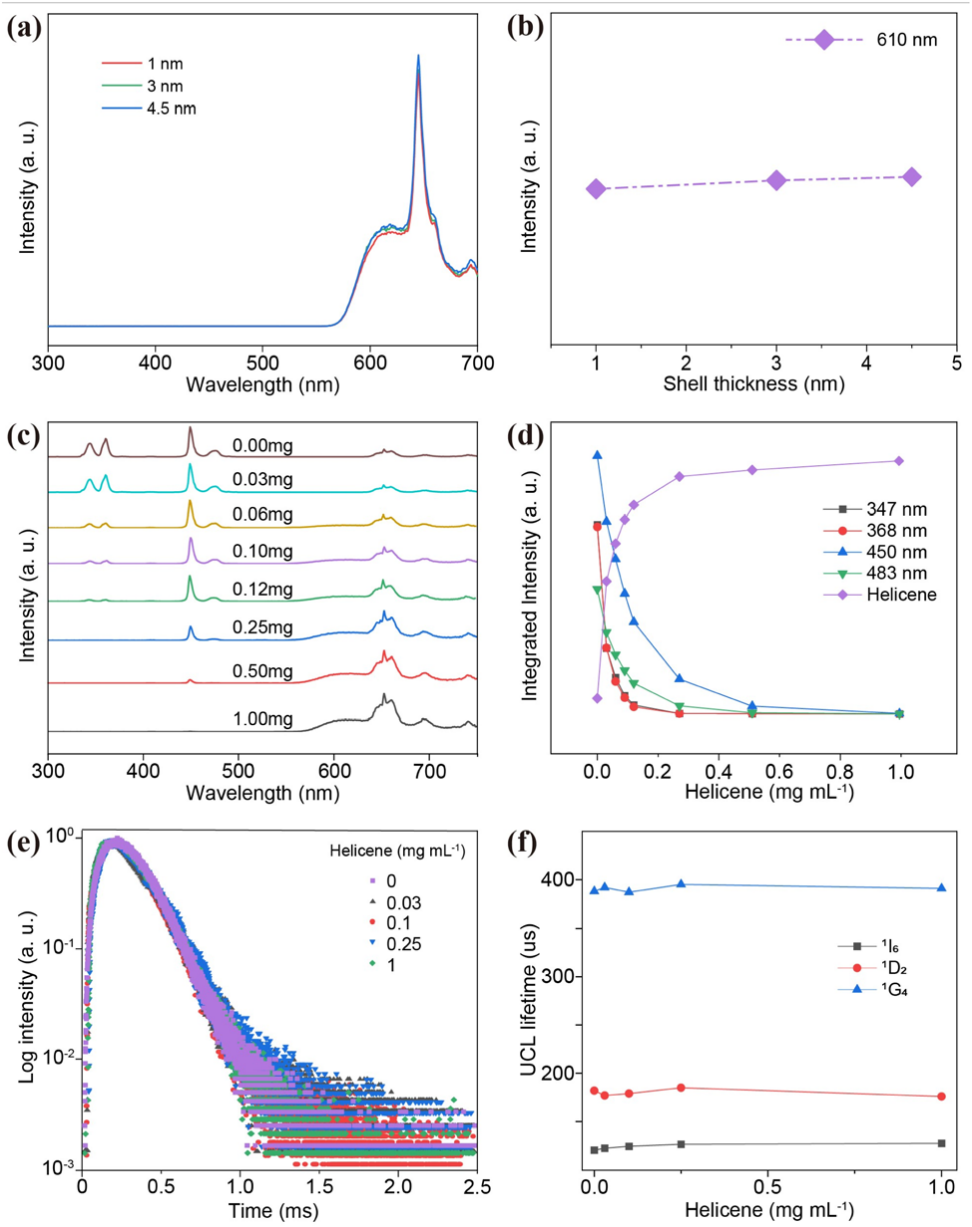
(a) UCL spectra of NPs-helicene with different NaLuF_4_ shell thicknesses (1 nm, 3 nm, and 4.5 nm) under 980 nm excitation at a power density of 22 W cm^−2^. (b) Integrated intensities of NPs-helicene at 610 nm, plotted against different shell thicknesses, derived from panel (a). (c) Concentration-dependent UCL spectra of NPs-helicene with a NP concentration of 40 mg mL^−1^ under 980 nm excitation at a power density of 22 W cm^−2^. (d) Integrated intensities of the NP emission peaks at 347 nm, 362 nm, 450 nm, and 483 nm, alongside the helicene emission peak at 610 nm, plotted against different helicene concentrations, derived from panel (c). (e) UCL decay curves of ^1^D_2_ by monitoring the emission of NPs at 450 nm of NPs-helicene with different helicene concentrations. (f) UCL lifetimes of the ^1^I_6_, ^1^D_2_, and ^1^G_4_ states of Tm^3+^ ions of NPs-helicene at varying helicene concentrations.

We deduced that the energy transfer mechanism in this system is a radiative energy transfer process rather than a non-radiative Förster resonance energy transfer (FRET) process. This distinction arises from the presence of an inert shell on the NPs, which acts as a physical barrier between the energy donor and acceptor. To elucidate the influence of inert shell thickness on luminescence, we synthesized core–shell NPs with varying shell thicknesses (1, 3, and 4.5 nm) (ESI, Fig. S16†). Under 980 nm excitation, increasing the inert shell thickness had minimal impact on the UCL of NPs-helicene, with almost no change observed as the shell thickness increased (Fig. 3a and b). This behavior contrasts sharply with the distance-dependent quenching expected in a FRET process, supporting our hypothesis. To further validate this hypothesis regarding the energy transfer process, we investigated the luminescence response as a function of helicene concentration. Under excitation at 980 nm, the red emission intensity of helicene at 610 nm gradually increases with an increasing concentration. The enhancement in red-light emission is accompanied by a concurrent decrease in the emission intensity of Tm^3+^ in the UV-blue regions, indicating energy transfer from the NPs to helicene (Fig. 3c). Notably, the reduction in Tm^3+^ emissions at 347 nm (^1^I_6_ → ^3^F_4_) and 362 nm (^1^D_2_ → ^3^H_6_) occurs at a significantly faster rate than at 450 nm (^1^D_2_ → ^3^F_4_) and 483 nm (^1^G_4_ → ^3^H_6_) (Fig. 3d). These observations can be attributed to the higher absorbance of helicene in the UV region compared to the blue region (ESI, Fig. S7†). It is important to emphasize that a non-radiative FRET process would simultaneously quench the emissions from the ^1^I_6_, ^1^D_2_ and ^1^G_4_ levels of Tm^3+^.^46^ However, the observed variations in emission intensities across different peaks suggest that the energy transfer from the NPs to helicene may follow a radiative reabsorption mechanism. This radiative transfer process was further supported by the UCL decay curves of Tm^3+ 1^I_6_ → ^3^F_4_, ^1^D_2_ → ^3^H_6_, ^1^D_2_ → ^3^F_4_, and ^1^G_4_ → ^3^H_6_ transitions in NPs and NPs-helicene, under the excitation of a 980 nm pulsed laser (Fig. 3e and f and ESI, Fig. S17†).^47^ Importantly, the UCL lifetimes of Tm^3+^ remain unchanged on varying the concentration of helicene, indicating that the lifetimes of the sensitizer are independent of the activator concentration. These observations align with our previous findings,^42^ wherein the lifetimes of the ^1^I_6_, ^1^D_2_, and ^1^G_4_ states of Tm^3+^ remained constant within the millisecond range in the NPs-perovskite systems, regardless of the molar ratio of NPs to perovskite. Collectively, these results provide strong evidence that the energy transfer from NPs to helicene occurs *via* a radiative reabsorption mechanism rather than a non-radiative FRET process.

### Biological sensing application

**Fig. 4:**
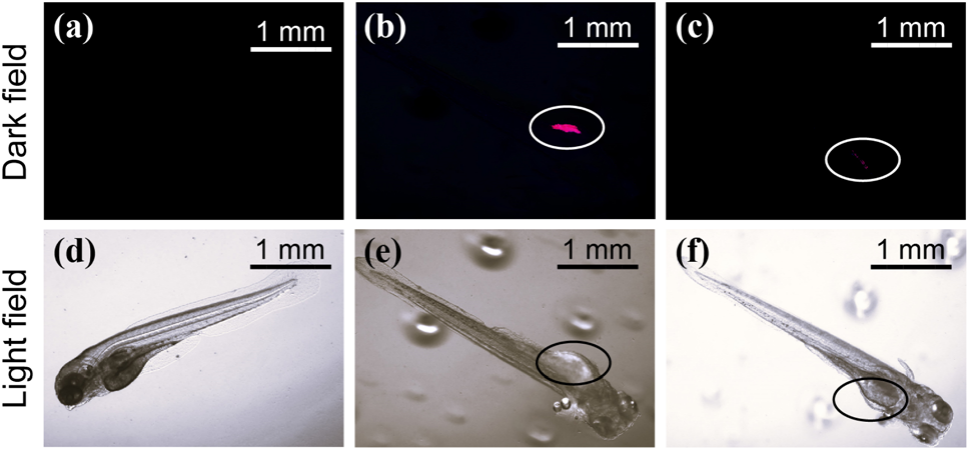
Fluorescence images of zebrafish (a and d) control group, (b and e) zebrafish stained with NPs-helicene, and (c and f) NPs-helicene + Fe^2+^ (*λ*_ex_ = 980 nm). The location marked with a circle is the stomach of zebrafish.

We successfully achieved UCL in helicene through NP sensitization, shifting its excitation wavelength from the UV-blue region to the NIR region. This transition enhances the spatial resolution and tissue penetration depth of helicene, broadening its potential applications in biological imaging. To demonstrate this, we conducted a penetration depth comparison using pig skin tissue (0.12 mm thick) (ESI, Fig. S18†). Under 980 nm laser excitation, NPs-helicene emitted red light, whereas 365 nm excitation failed to penetrate the tissue effectively. This highlights the advantage of NIR excitation for improved tissue penetration. Additionally, fluorescence probe selectivity experiments revealed that helicene exhibits significant fluorescence quenching in the presence of Fe^2+^, while other common metal ions do not interfere with its luminescence (*λ*_ex_ = 365 nm) (ESI, Fig. S19 and S20†). This selectivity was retained in the NPs-helicene system (*λ*_ex_ = 980 nm) (ESI, Fig. S21†). Building on these findings, we designed a simple application of NPs-helicene as a NIR-responsive probe in zebrafish embryos. Fig. 4a and d show control zebrafish without NPs-helicene or Fe^2+^. Upon absorption of NPs-helicene, bright red emission was observed in the stomach region under 980 nm excitation (Fig. 4b and e). The addition of Fe^2+^ resulted in significant quenching of the red emission under the same excitation conditions (Fig. 4c and f), consistent with *in vitro* observations. This experiment exemplifies the potential of NPs-helicene as a NIR-responsive fluorescent probe for biological sensing.

### Generality of the proposed sensitization strategy

**Fig. 5:**
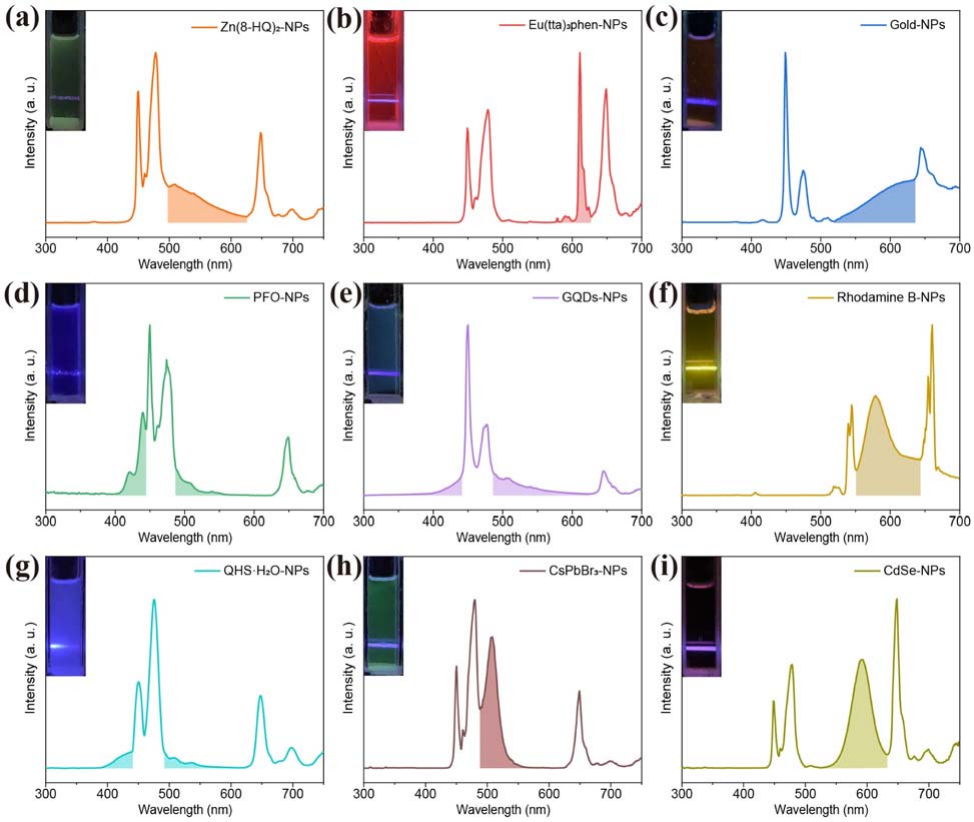
UCL spectra of (a) Zn(8-HQ)_2_, (b) Eu(tta)_3_phen, (c) gold clusters, (d) poly(9,9-dioctylfluorene-2,7-diyl) (PFO), (e) graphene quantum dots (GQDs), (g) quinine hemisulfate monohydrate (QHS·H_2_O), (h) CsPbBr_3_ quantum dots (QDs), and (i) CdSe QDs, sensitized with NaLuF_4_:33%Gd/49%Yb/0.5%Tm@NaLuF_4_ core–shell NPs. (f) UCL spectrum of rhodamine B, sensitized using NaYF_4_:18%Yb/2%Er@NaYF_4_ core–shell NPs. All emissions were excited at a 980 nm wavelength with a power density of 22 W cm^−2^.

Based on the above design strategy, we further explored UCL of various activators including transition metal complexes, lanthanide complexes, metal nanoclusters, conjugated polymers, nanoscale carbon allotropes, fluorescent dyes, and quantum dots, through sensitization with lanthanide-doped NPs (ESI, Fig. S22 and S23†). Remarkably, for the NPs-Zn(8-HQ)_2_ composite, excitation with a 980 nm laser resulted in the observation of an emission peak at 510 nm, which can be attributed to the characteristic emission of the transition metal complex (Fig. 5a). Similarly, the lanthanide complex (Eu(tta)_3_phen) can also be sensitized using NPs, producing its characteristic emissions centered at 589 and 610 nm (Fig. 5b). Additionally, NP-sensitized gold nanoclusters exhibited an emission peak at 630 nm (Fig. 5c). The UCL emissions of a conjugated polymer (PFO) were also observed at 420 nm and 440 nm when sensitized using NPs (Fig. 5d). Similarly, typical UCL emissions from carbon allotropes, such as graphene quantum dots (GQDs), were detected at 460 nm and from fluorescent dyes, such as rhodamine B (560 nm) and quinine hemisulfate monohydrate (QHS·H_2_O) (450 nm), and from quantum dots including perovskite quantum dots (CsPbBr_3_ at 510 nm) and semiconductor quantum dots (CdSe at 590 nm) were detected under NIR excitation through NP-sensitization (Fig. 5e–i). It is noteworthy that no emission from the composite systems was detected in the absence of NPs under identical conditions. Additionally, using eqn (1), we calculated the energy transfer efficiency for these composite systems (ESI, Table S2†). Notably, NPs-Eu(tta)_3_phen, NPs-PFO, and NPs-GQDs all demonstrated energy transfer efficiencies exceeding 99.9%.

The proposed radiative energy transfer nanosystem can also be extended to the sensitization of rhodamine B by employing different excitation wavelengths of lanthanide-doped NPs as energy donors. For example, through sensitization with NaErF_4_@NaLuF_4_ NPs and NaGdF_4_:20%Yb/2%Er@NaGdF_4_:10%Nd/10%Yb NPs under 1532 nm and 808 nm excitations, tunable upconverted emissions were detected in rhodamine B (ESI, Fig. S24†).^48,49^ Furthermore, we demonstrated that the NP-sensitized rhodamine B can be fabricated as a film by casting it onto a glass substrate, from which intense red upconversion emissions were realized under CW diode laser excitation (ESI, Fig. S25†). Taking together, these results demonstrate that NPs serve as versatile sensitizers for photon upconversion across a wide range of activators, with the crucial advantage of operating under NIR light excitation.

## Conclusions

In conclusion, we have presented a strategy to boost UCL efficiency across a wide range of luminescent nanomaterials (chiroptical materials, transition metal complexes, lanthanide complexes, metal nanoclusters, conjugated polymers, nanoscale carbon allotropes, fluorescent dyes, and quantum dots) through sensitization with lanthanide-doped NPs. These lanthanide-doped NPs have been demonstrated to extend the NIR responsivity of optical nanomaterials by converting NIR photons into UV or visible photons. Furthermore, multicolor UCL emissions with tunable wavelengths, lifetimes, and polarization from optical nanomaterials are achieved *via* a radiative energy transfer upconversion process from NPs to these nanomaterials. The integration of luminescent nanomaterials with lanthanide-doped NPs not only imparts NIR responsivity to the luminescent nanomaterials but also introduces new, multi-dimensional tunable emission spectra for photon upconversion. This strategy overcomes the inherent limitations of individual optical nanomaterials and NPs, opening new avenues for materials and device engineering in advanced optoelectronic applications.

## Ethical statement

All animal experiment procedures were approved by the Ethics Committee for Animal Experiments of Jiangsu University and performed in agreement with the relevant laws and institutional guidelines.

## Data availability

The data supporting this article have been included as part of the ESI.†

## Author contributions

J. L.: investigation, methodology, writing – original draft. J. S.: investigation, methodology. Y. L., X. Y., J. Z., W. Z., T. H., and G. F.: concept discussion, writing – review & editing. W. Z. and X. Cheng: writing – review & editing, supervision. X. Chen: supervision, funding acquisition, project administration, writing – review & editing.

## Conflicts of interest

There are no conflicts to declare.

## Supplementary Material

SC-016-D5SC00937E-s001
