# Supplementary material for: Boosting near-infrared-triggered photon upconversion in optical nanomaterials via lanthanide-doped nanoparticle sensitization
Source: Chem Sci. 2025 Apr 10;16(20):8820–6. doi: 10.1039/d5sc00937e (PMC11998938; doi:10.1039/d5sc00937e)
Supplement: SC-016-D5SC00937E-s001 [file SC-016-D5SC00937E-s001.pdf]

Supplementary Information (SI) for Chemical Science.
This journal is © The Royal Society of Chemistry 2025

Supporting Information

Boosting near-infrared-triggered photon upconversion in optical nanomaterials via lanthanide-doped nanoparticle sensitization

Jiangshan Luo,‡[a] Junjian Shen,‡[c] Xingwen Cheng,*[a,d] Yan Liu,[b] Xiulian Yin,[a] Tianxi Hu,[d] Guangxin Fan,[d] Jianming Zhang,[a] Wei Zheng*[b] and Xueyuan Chen*[b]

[a.] *Institute of Quantum and Sustainable Technology (IQST), School of Chemistry and Chemical Engineering, Jiangsu University, Zhenjiang, Jiangsu, 212013 China.*

E-mail: chengxw@ujs.edu.cn (X. Cheng)

[b.] *State Key Laboratory of Structural Chemistry and Fujian Key Laboratory of Nanomaterials, Fujian Institute of Research on the Structure of Matter, Chinese Academy of Sciences, Fuzhou, Fujian 350002, China.*

E-mail: zhengwei@fjirsm.ac.cn (W. Zheng), xchen@fjirsm.ac.cn (X. Chen)

[c.] *School of the Environment and Safety Engineering, Jiangsu University Zhenjiang, Jiangsu, 212013, China.*

[d.] *Yongkang Hardware Technician College, Yongkang, Zhejiang, 321300, China.*

‡ These authors contributed equally to this work.

Experimental Section

Chemicals and Materials: Lu_2O_3 (99.99%), Y_2O_3 (99.99%), Gd_2O_3 (99.99%), Tm_2O_3 (99.99%), Yb_2O_3 (99.99%), $Na(CF_3COO)$, cyclohexane, hexane, ethanol and dichloromethane were purchased from Macklin (Shanghai, China). Oleic acid (OA, 90%) and 1-octadecene (ODE, 90%) were purchased from Sigma-Aldrich (Shanghai, China). Oleylamine (OM, 80%-90%) was purchased from Aladdin (Shanghai, China). All the chemical reagents were used as received without further purification. $Ln(CF_3COO)_3$ were prepared by the literature method.[1]

Synthesis of $NaLuF_4$:33%Gd/49%Yb/0.5%Tm core NPs: In a typical synthesis of the core nanoparticles (NPs), 0.5 mmol of $Ln(CF_3COO)_3$ (Ln = Lu, Gd, Yb, Tm) and 0.75 mmol of $Na(CF_3COO)$ were added to a 50 mL flask containing OA (5.9356 g), OM (2.9627 g) and ODE (3.0197 g). The mixture was then heated to 140 °C under magnetic stirring in an N_2 atmosphere for 15 minutes. Afterward, the transparent solution was heated to 310 °C with vigorously stirring for 60 minutes before being allowed to cool naturally to room temperature (RT). The product was washed twice with ethanol and cyclohexane, and subsequently redispersed in cyclohexane.

Synthesis of $NaLuF_4$:33%Gd/49%Yb/0.5%Tm@$NaLuF_4$ core-shell NPs: For the core-shell structure synthesis, the core NPs prepared in the previous step were added to a 50 mL flask containing OA (6.2954 g) and ODE (5.5626 g). The mixture was heated to 90 °C under magnetic stirring in an N_2 atmosphere, then cooled to RT. Next, 0.5 mmol $Ln(CF_3COO)_3$ and 0.5 mmol $Na(CF_3COO)$ were added to the flask, and the mixture was heated to 120 °C under magnetic stirring for 15 minutes. The solution was then heated to 300 °C with vigorously stirring for 60 minutes and allowed to cool naturally to RT. The resulting product was washed twice with ethanol and cyclohexane, and finally redispersed in dichloromethane.

Zebrafish fluorescence imaging: For bioimaging *in vivo*, 5-day-old zebrafishes were prepared. Zebrafishes were fed with 10 μM of NPs-helicene probe in E3 embryo media (15 mM NaCl, 0.5 mM KCl, 1 mM $MgSO_4$, 1 mM $CaCl_2$, 0.15 mM KH_2PO_4, 0.05 mM Na_2HPO_4, 0.7 mM $NaHCO_3$, 0.0001% methylene blue; pH 7.5) at 28 °C for 6 hours. Then added Fe^{2+} (50 μM) for 30 min. All the fishes were terminally anaesthetized using MS-222, and images were carried out on a confocal microscope.

Nanoparticle–molecule film fabrication: Samples were fabricated on square glass substrates. In a typical experiment, the glass substrates were first cleaned by sequential sonication in isopropanol and acetone, followed by treatment with oxygen plasma for 10 minutes. The resulting nanoparticle–molecule mixed solution was drop-cast onto the glass substrates to form blend films.

Theoretical Calculations: Density functional theory (DFT) calculations were performed using the Gaussian 09 software package. The geometries, molecular orbitals, and molecular orbital (MO) energies were calculated at the B3LYP-D3/6-31G(d, p) level. The UV-vis absorption and circular dichroism (CD) spectra were simulated by time-dependent DFT calculations at the same level of theory.

Structural and Optical Characterization: Powder X-ray powder diffraction (XRD) patterns of the samples were obtained using an X-ray diffractometer (D8 ADVANCE, Bruker) with Cu Kα1 radiation (λ = 0.154187 nm). Low-resolution and high-resolution transmission electron microscopy (TEM) measurements were carried on a Talos F200X G2 (Thermo Fisher Scientific) with the energy-dispersive X-ray spectrum (EDS). Optical absorption spectra of various luminescent materials, including chiroptical materials, transition metal complexes, lanthanide complexes, metal

nanoclusters, conjugated polymers, nanoscale carbon allotropes, fluorescent dyes, quantum dots were recorded in transmission mode using a Shimadzu UV-3600i Plus 950 UV-Vis-NIR spectrometer. Photoluminescence (PL) excitation spectra, emission spectra, and PL decay measurements were performed using an FS5 spectrometer (Edinburgh) equipped with both a continuous xenon lamp (150 W) and a pulsed flash lamp. Upconversion luminescence (UCL) emission spectra were measured under 980 nm excitation at a power density of 22 W/cm^2 using a continuous-wave diode laser. UCL lifetimes were determined using a customized UV to mid-infrared steady-state and phosphorescence lifetime spectrometer (FSP920-C, Edinburgh) equipped with a digital oscilloscope (TDS3052B, Tektronix) and a tunable mid-band Optical Parametric Oscillator pulsed laser as the excitation source (410-2400 nm, 10 Hz, pulse width ≤5 ns, Vibrant 355II, OPOTEK). Separation of the chiral isomers of helicene was performed on a Shimadzu LC-20AT instrument equipped with a CHIRALPAK IE-3 (IE30CE-XB011) column (0.46 cm I.D. × 25 cm L). The photos of the zebrafishes were taken using an inverted microscope (IXplore IX85, Olympus). PL photographs were taken using an iPhone 11 cell phone without any optical filters. A standard tungsten lamp was employed to correct the optical response of the instrument. All the spectral data were acquired at RT and corrected for the spectral response of both the spectrometer and the integrating sphere.

Table S1 Selected TD-DFT (B3LYP-D3/6-31G(d, p)) calculated energies, oscillator strength and compositions of major electronic transitions of helicene.

excited state	energy (eV)	wavelength (nm)	oscillator strength (f)	description
1	2.1942	565.06	0.1381	H→L (0.98)
2	2.4104	514.37	0.1054	H-1→L (0.97)
3	2.6457	468.62	0.0028	H-2→L (0.95)<br>H-4→L (0.03)
4	2.6561	466.78	0.2157	H-3→L (0.90)<br>H→L+2 (0.05)
5	2.7150	456.66	0.0136	H-6→L (0.17)<br>H-4→L (0.79)<br>H-2→L (0.03)
6	2.7661	448.23	0.0001	H-5→L (0.96)
7	2.7820	445.66	0.0030	H-6→L (0.80)<br>H-4→L+3 (0.17)
8	2.9467	420.75	0.0008	H-7→L (0.98)
9	2.9479	420.58	0.0008	H-8→L (0.97)
10	3.1210	397.25	0.0077	H-9→L (0.89)<br>H→L+1 (0.07)

Table S2 The energy transfer efficiency between NPs and different A (A = activators).

A:	$Zn(8\text{-}HQ)_2$	$Eu(tta)_3phen$	gold clusters	PFO	GQDs	rhodamine B	QHS $\cdot H_2O$	$CsPbBr_3$ QDs	CdSe QDs
η^{ETE}:	52.5%	>99.9%	58.4%	>99.9%	>99.9%	40.7%	>99.9%	45.2%	89.5%

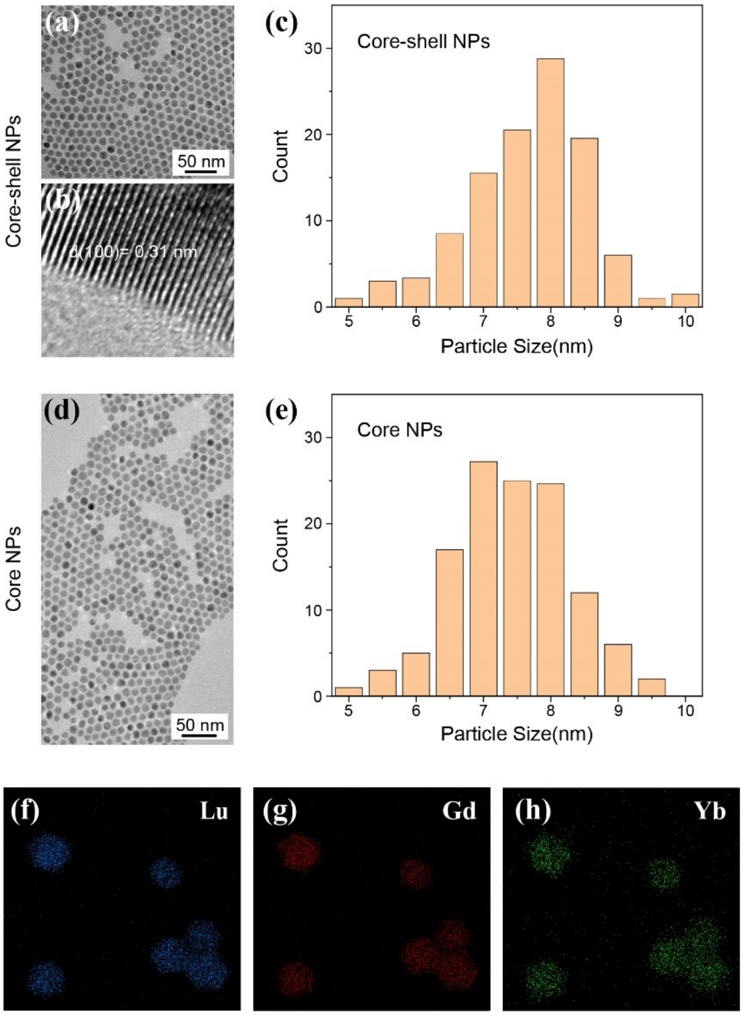

Figure S1. a) Low-resolution and b) high-resolution TEM images of $NaLuF_4$:33%Gd/49%Yb/0.5%Tm@$NaLuF_4$ core-shell NPs. c) Particle size distributions of core-shell NPs. d) TEM image of $NaLuF_4$:33%Gd/49%Yb/0.5%Tm core NPs. e) Particle size distributions of the core NPs. EDS element mappings of f) Lu^{3+}, g) Gd^{3+}, and h) Yb^{3+} ions in $NaLuF_4$: Yb/Gd/Tm@$NaLuF_4$ NPs. The TEM images revealed that the core-shell and core NPs were approximately spherical, with their sizes of 8 nm and 7 nm, respectively. The size distributions were determined by randomly analyzing 200 particles. High-resolution TEM showed that the NPs exhibited a single crystalline hexagonal structure. EDS confirmed the presence of lanthanide elements Lu, Gd, and Yb in the core-shell NPs.

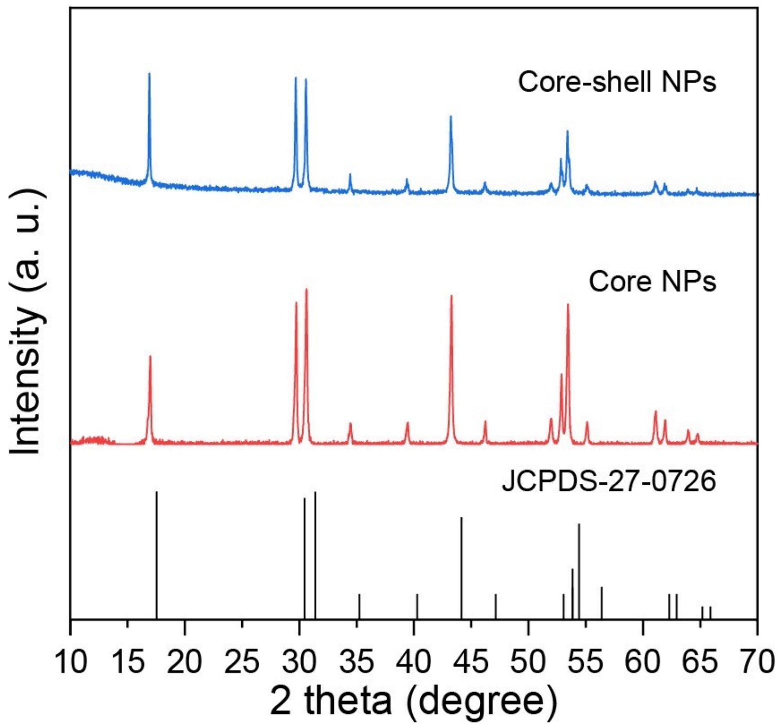

Figure S2. Powder XRD patterns of $NaLuF_4$:33%Gd/49%Yb/0.5%Tm core NPs and $NaLuF_4$:33%Gd/49%Yb/0.5%Tm@$NaLuF_4$ core-shell NPs. All the diffraction peaks of the NPs match well with the standard pattern of $NaLuF_4$ (JCPDS No. 27-0726), indicating that the core and core-shell NPs exhibit a pure phase and high crystallinity.

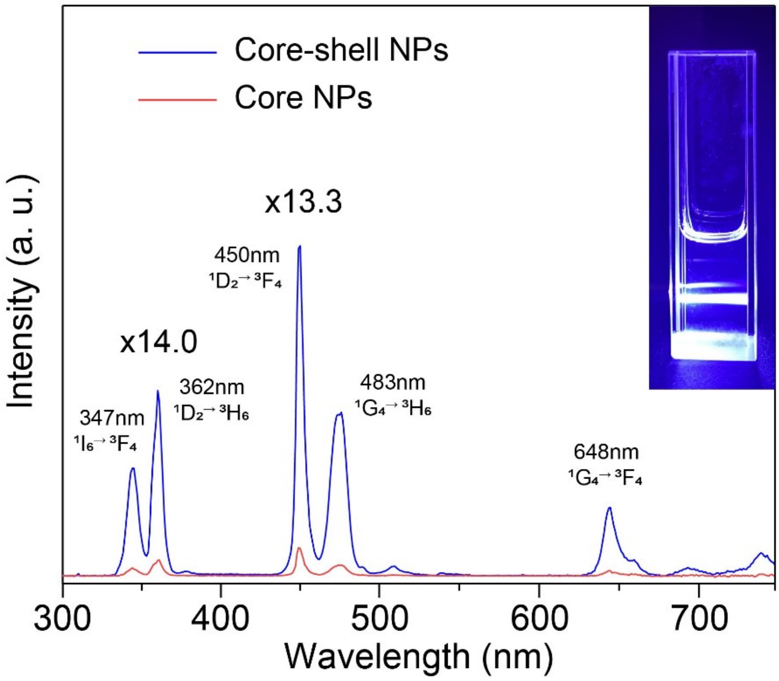

Figure S3. UCL spectra and corresponding photographs of $NaLuF_4$:33%Gd/49%Yb/0.5%Tm@$NaLuF_4$ core-shell NPs (blue) and $NaLuF_4$:33%Gd/49%Yb/0.5%Tm core NPs (red) under continuous-wave diode laser excitation at 980 nm. Yb^{3+} ions serve as sensitizers, absorbing near-infrared light at 980 nm, while Tm^{3+} ions function as activators, leading to strong upconversion emissions. Under 980 nm excitation, emissions at 347 nm ($^1I_6 \rightarrow {}^3F_4$) and 362 nm ($^1D_2 \rightarrow {}^3H_6$) fall within the violet region, while emissions at 450 nm ($^1D_2 \rightarrow {}^3F_4$) and 483 nm ($^1G_4 \rightarrow {}^3H_6$) are situated in the blue region, and emissions at 648 nm ($^1G_4 \rightarrow {}^3F_4$) belong to the red region. Notably, after coating with an inert $NaLuF_4$ shell, emissions at 362 nm (UV region) and 450 nm (blue region) increased by 14.0 and 13.3 times, respectively.

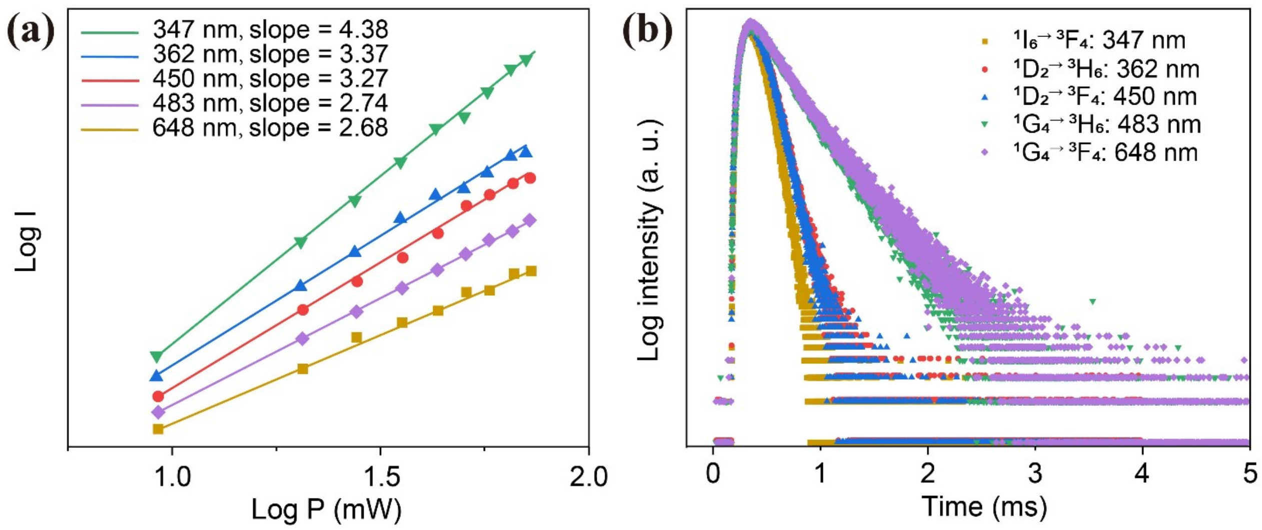

Figure S4. a) Power density dependence of Tm^{3+} emissions at 347 nm ($^1I_6 \rightarrow {}^3F_4$), 362 nm ($^1D_2 \rightarrow {}^3H_6$), 450 nm ($^1D_2 \rightarrow {}^3F_4$), 483 nm ($^1G_4 \rightarrow {}^3H_6$), and 648 nm ($^1G_4 \rightarrow {}^3F_4$), indicating a five-, four-, and three-photon population processes in the $NaLuF_4$:33%Gd/49%Yb/0.5%Tm@ $NaLuF_4$ core-shell NPs under excitation of a 980-nm diode laser. b) UCL decay curves from the $^1I_6$, $^1D_2$, and $^1G_4$ levels of Tm^{3+} by monitoring the emissions at 347, 362, 450, 483, and 648 nm, respectively, in $NaLuF_4$:33%Gd/49%Yb/0.5%Tm@$NaLuF_4$ core-shell NPs under 980 nm excitation. By fitting the decay curves with a single-exponential model, the UCL lifetimes for the $^1I_6 \rightarrow {}^3F_4$, $^1D_2 \rightarrow {}^3H_6$, $^1D_2 \rightarrow {}^3F_4$, $^1G_4 \rightarrow {}^3H_6$, and $^1G_4 \rightarrow {}^3F_4$ transitions of Tm^{3+} were determined to be 131, 175, 180, 391 and 394 μs, respectively.

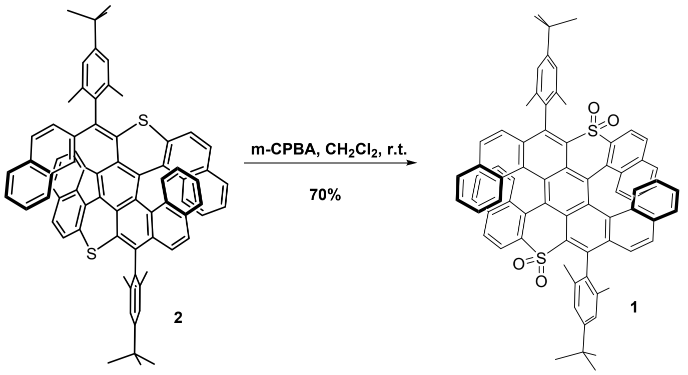

Figure S5. The synthesis route of helicene (compound 1). Precursor (compound 2) was synthesized according to the reference.[2] To a suspension of precursor (30 mg, 0.03 mmol) in a solvent of CH_2Cl_2 (10 mL) was added m-chloroperbenzoic acid (m-CPBA, 26 mg, 0.15 mmol). The mixture was stirred for 12 h at room temperature. The organic solvent was removed under reduced pressure, and then the crude product was purified by column chromatography on silica gel (eluent: hexane/CH_2Cl_2 = 3:1) to give helicene as an orange-red solid in 70% yield.

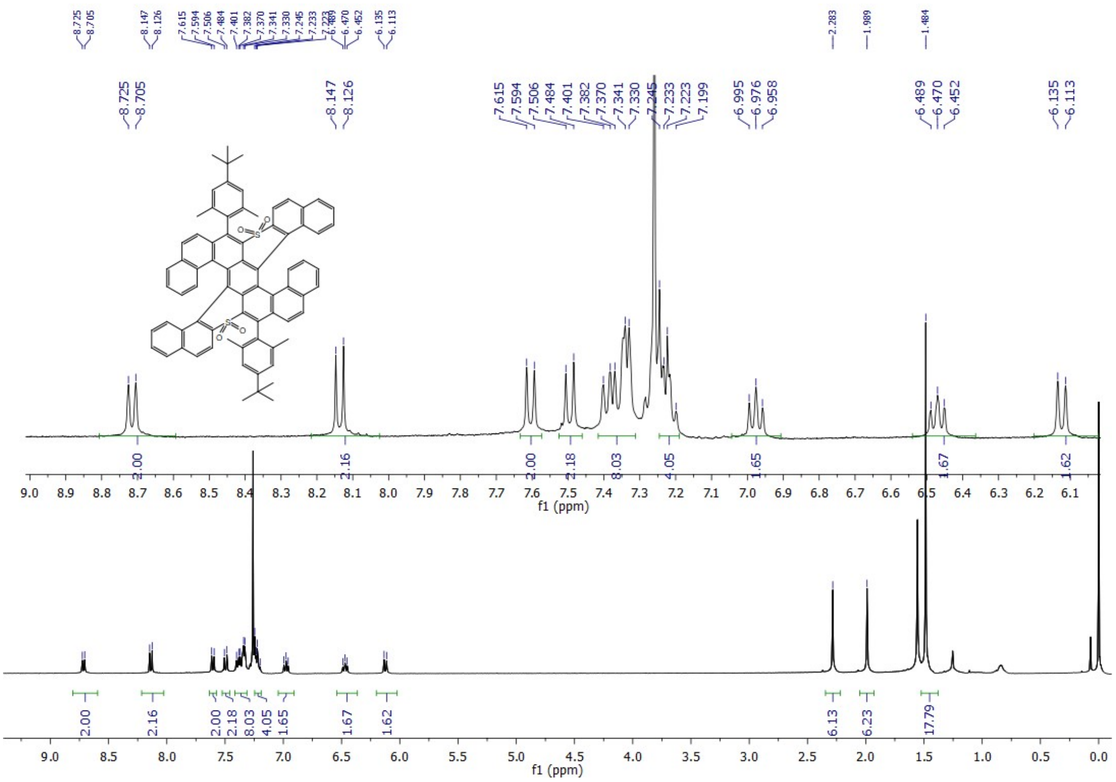

Figure S6. ^{1}H NMR spectrum of helicene (400 MHz, $CDCl_3$). ^{1}H NMR (400 MHz, $CDCl_3$): δ (ppm) 8.715 (d, J = 8.0 Hz, 2H), 8.137 (d, J = 8.4 Hz, 2H), 7.605 (d, J = 8.4 Hz, 2H), 7.495 (d, J = 8.8 Hz, 2H), 7.30-7.42 (m, 8H), 7.15-7.25 (m, 4H), 6.976 (t, J = 8.0 Hz, 2H), 6.470 (t, J = 8.0 Hz, 2H), 6.124 (d, J = 8.8 Hz, 2H), 2.283 (s, 6H), 1.989 (s, 6H), 1.484 (s, 18H). The compound helicene was characterized and confirmed by ^{1}H NMR.

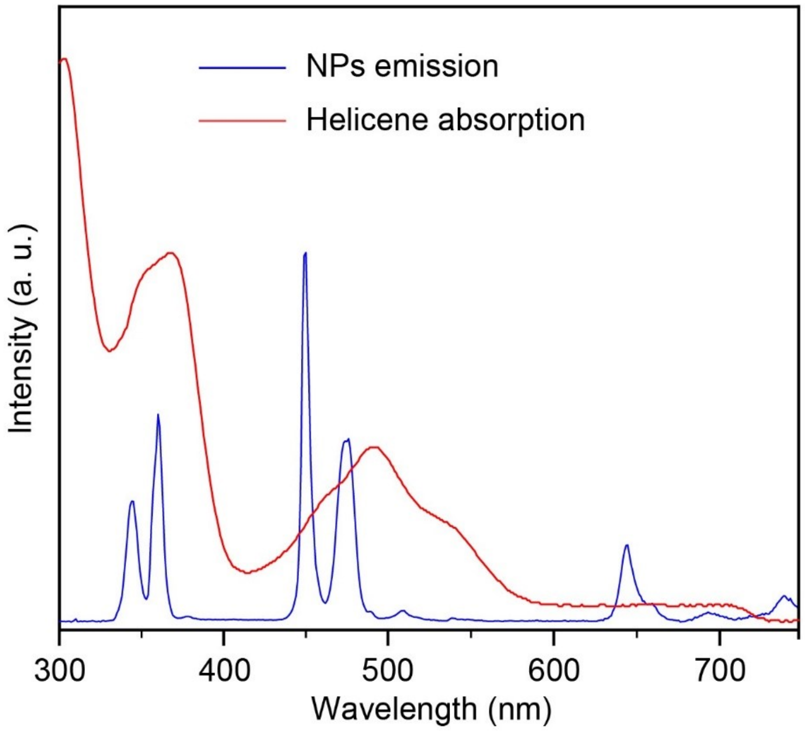

Figure S7. UCL spectra (λ_{ex} = 980 nm) of NPs and the absorption spectrum of helicene. The helicene shows stronger absorption in the violet region compared to the blue region, indicating that in the NPs-helicene system, helicene absorbs more violet light from Tm^{3+} than blue light.

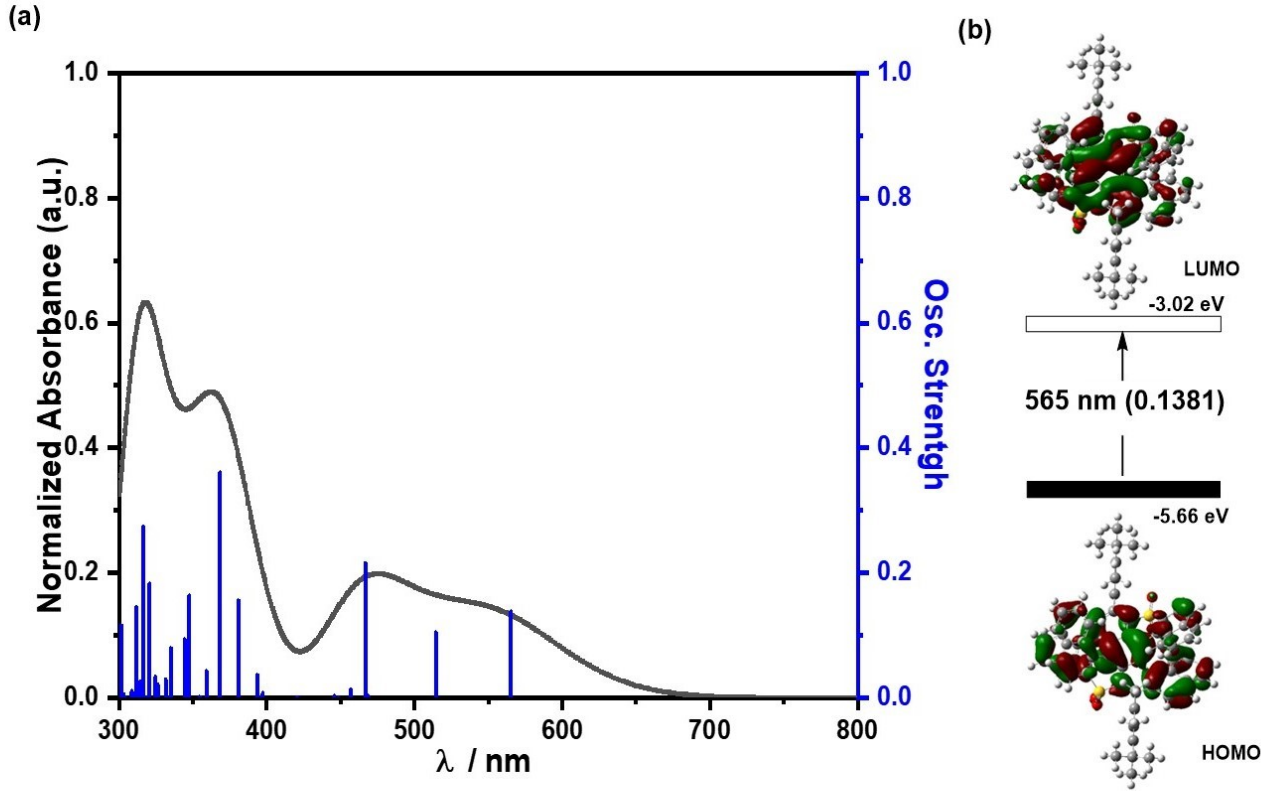

Figure S8. a) Simulated absorption spectrum (black line) and calculated oscillator strength (blue bar) of helicene at the B3LYP-D3/6-31G(d, p) level. b) Frontier molecular orbitals and energy diagrams of helicene calculated by (TD)DFT at the B3LYP-D3/6-31G(d, p) level. Values in parentheses represent the oscillator strengths (*f*). The geometries, molecular orbitals, and MO energies were calculated at the B3LYP-D3/6-31G(d, p) level.

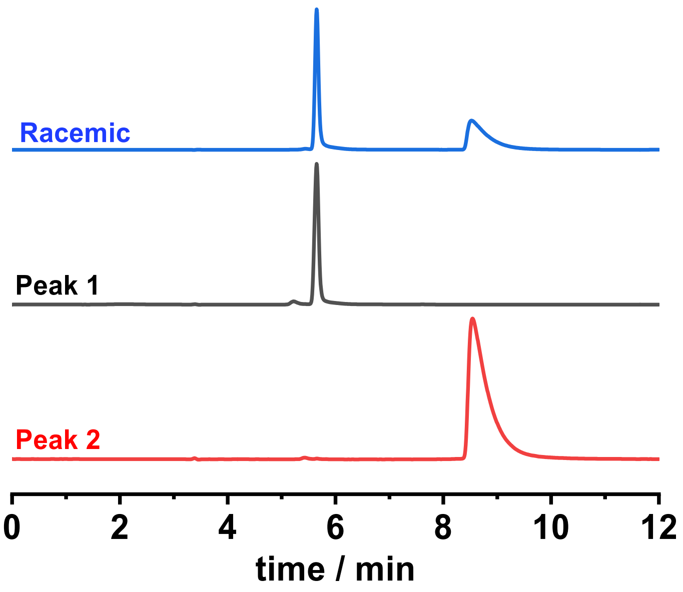

Figure S9. Chiral high performance liquid chromatography analysis of helicene. A mixture of Hexane/Dichloromethane = 45/55 (V/V) was used as the eluent with a flow rate of 1mL/min. The chiral isomers of helicene were successfully separated into two enantiomers (***M***,***M***)-**helicene** (peak 1) and (***P***,***P***)-**helicene** (peak 2).

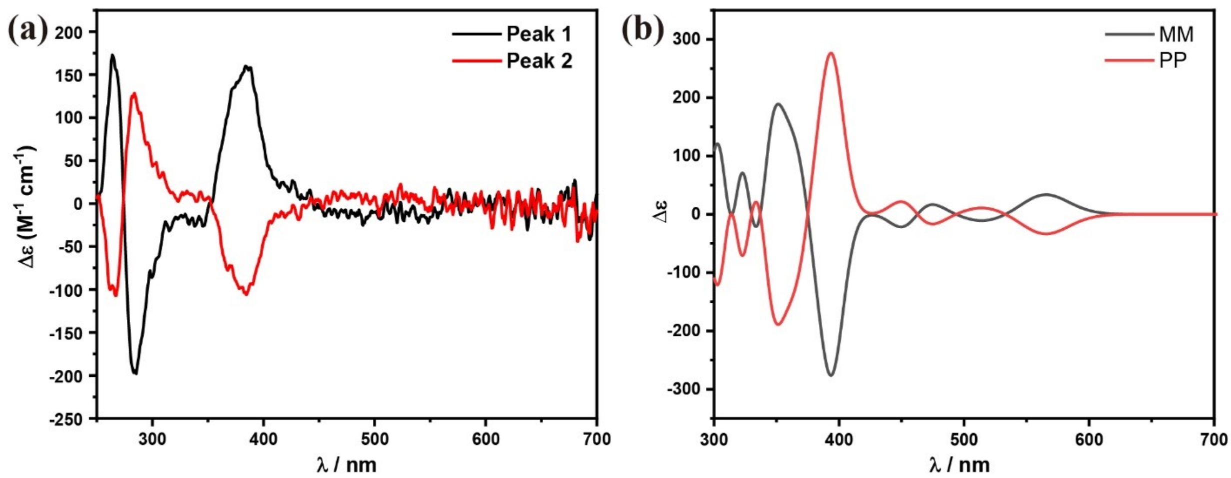

Figure S10. a) CD spectra of (*P*,*P*)-helicene (peak 1) and (*M*,*M*)-helicene (peak 2) in CH_2Cl_2 solution ($1{\times}10^{-5}$ M). b) Simulated CD spectra for the isomers of helicene at the B3LYP-D3/6-31G(d, p) level. The CD and simulated CD spectra reveals that the first peak obtained by chiral separation is (***M***,***M***)-**helicene**, while other peak corresponds to the (***P***,***P***)-**helicene**.

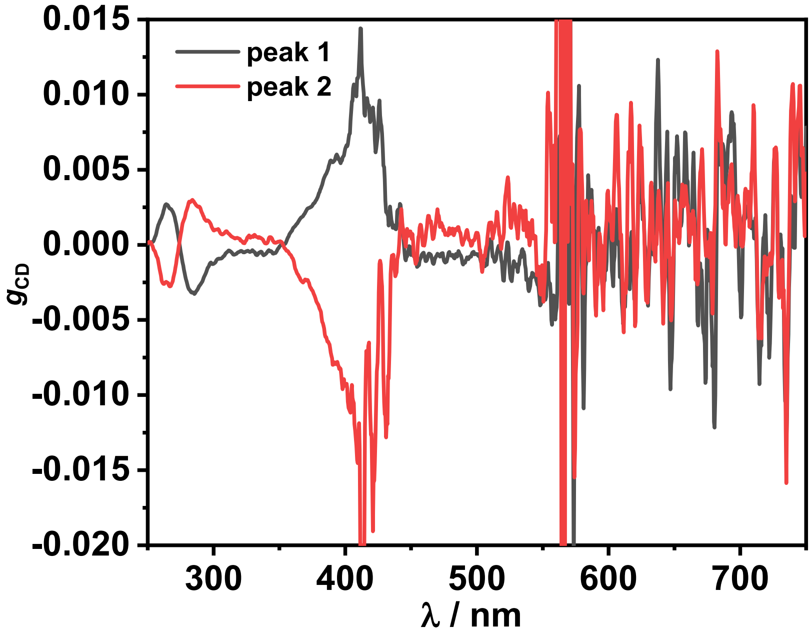

Figure S11. Absorption dissymmetry factors of (*P*,*P*)-helicene (peak 1) and (*M*,*M*)-helicene (peak 2) in CH_2Cl_2 solution (1×10^{-5} M). The dissymmetry factor is determined to be a maximum $|g_{abs}|$ (<0.015) at 410 nm.

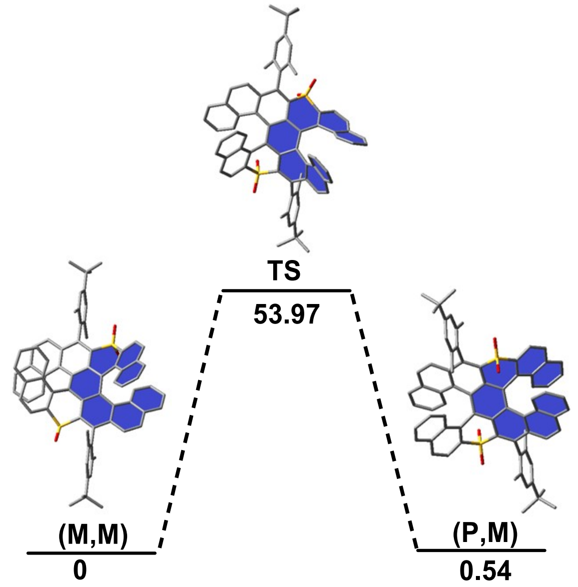

Figure S12. Isomerization process between (*M,M*)-helicene and (*P,M*)-helicene. The relative Gibbs free energy (unit: kcal mol^{-1}) was calculated at the B3LYP-D3/6-31G(d, p) level.

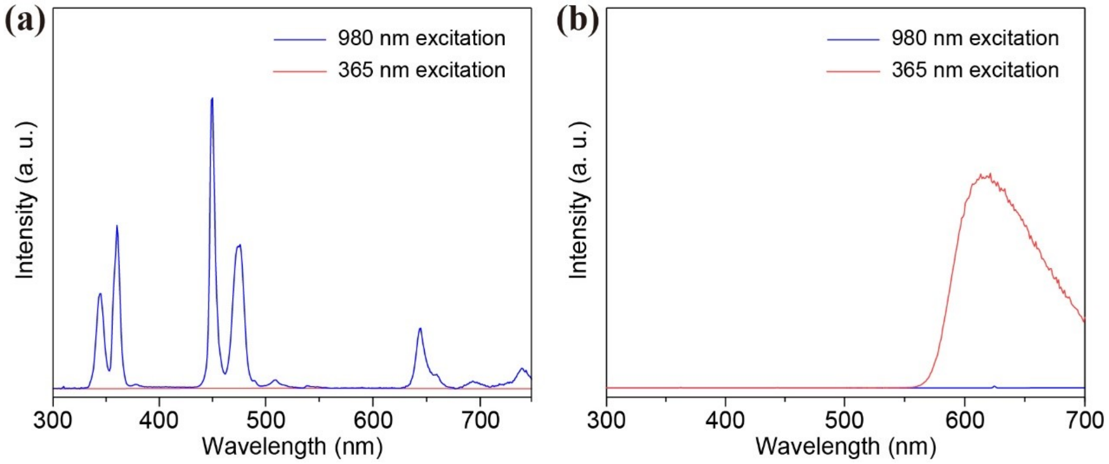

Figure S13. a) UCL spectra comparison of NPs under 980 nm and 365 nm excitation. b) PL spectra comparison of helicene under 980 nm and 365 nm excitation. This comparison experiment demonstrates that NPs do not exhibit any response to 365 nm excitation, and helicene show no reaction to 980 nm excitation.

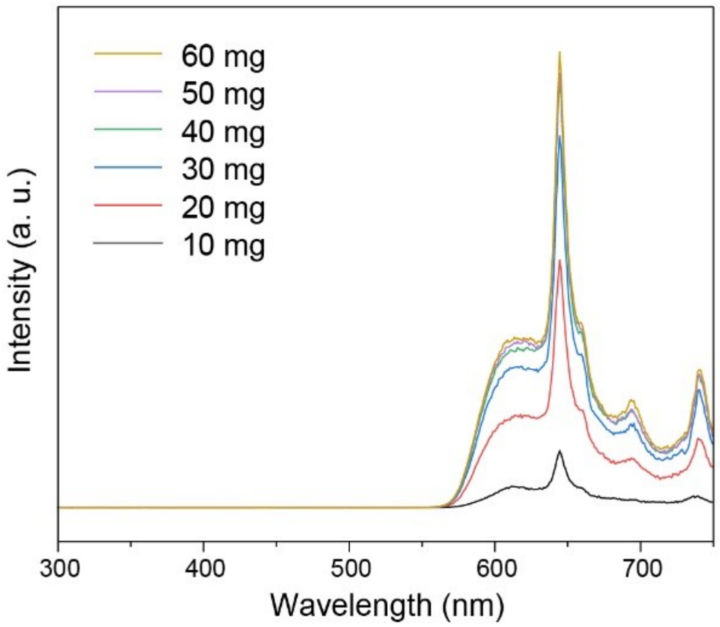

Figure S14. UCL spectra for NPs-helicene at a concentration of 1mg/mL for helicene and at varying NPs concentrations under 980 nm excitation. When the NP concentration exceeds 40 mg, no significant increase in the luminescent intensity of NPs-helicene is observed. Therefore, a 1:40 concentration ratio is considered optimal for NPs-helicene.

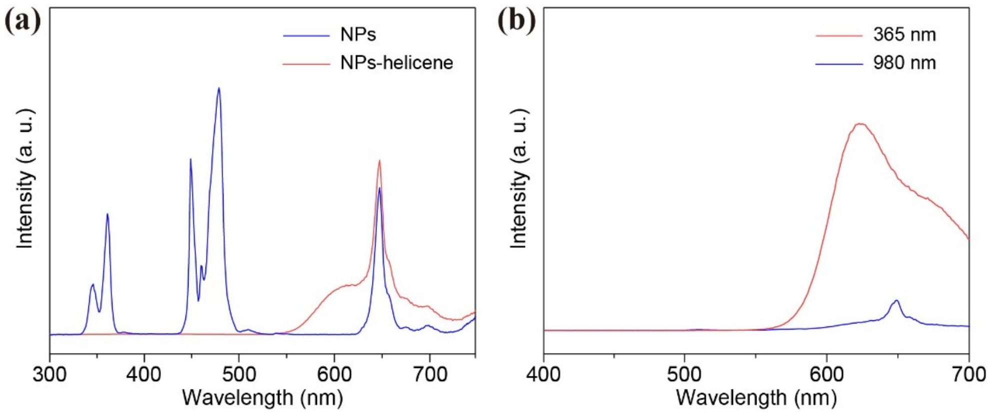

Figure S15. a) UCL spectra of NPs (blue) and NPs-helicene (red) under 980 nm excitation with the same power density of 22 W/cm^2. b) UCL spectra of NPs-helicene under 980 nm excitation and PL spectra of helicene under 365 nm excitation with the same power of 50 mW. NPs-helicene exhibits complete quenching of the blue-violet emission, with all emission concentrated in the red region. The spectra of both NPs and NPs-helicene under 980 nm excitation with the same power further confirm the high efficiency of this energy transfer process. Furthermore, we conducted a direct comparison between the UCL spectra of NPs-helicene under 980 nm excitation and PL spectra of helicene under 365 nm excitation, maintaining an identical excitation power (50 mW). The luminescence of helicene originates from a down-shifting process via direct excitation, whereas the spectrum of composite material results from a nonlinear multi-photon absorption process under near-infrared excitation. From the emission spectra, we can clearly observe that the NIR excitation of the composite material leads to distinct upconversion spectra. This strongly indicates the efficient sensitization process of lanthanide-doped NPs.

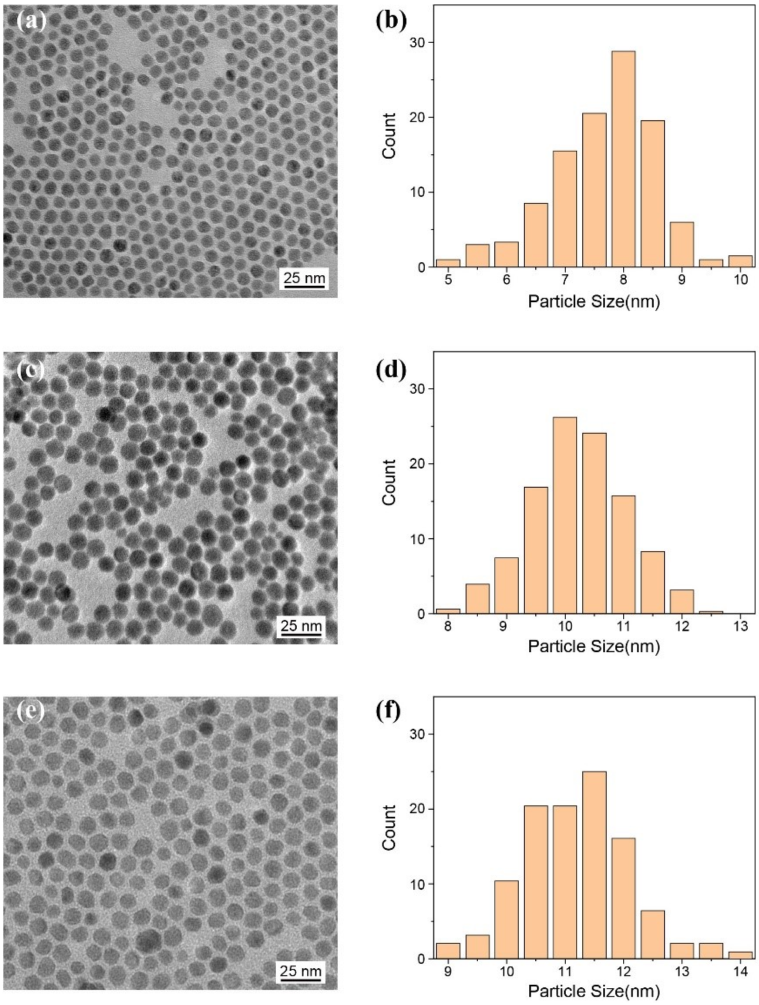

Figure S16. TEM images and corresponding size distribution of $NaLuF_4$:Gd/Yb/Tm@$NaLuF_4$ core-shell NPs with different shell thicknesses (1 nm, 3 nm, and 4.5 nm).

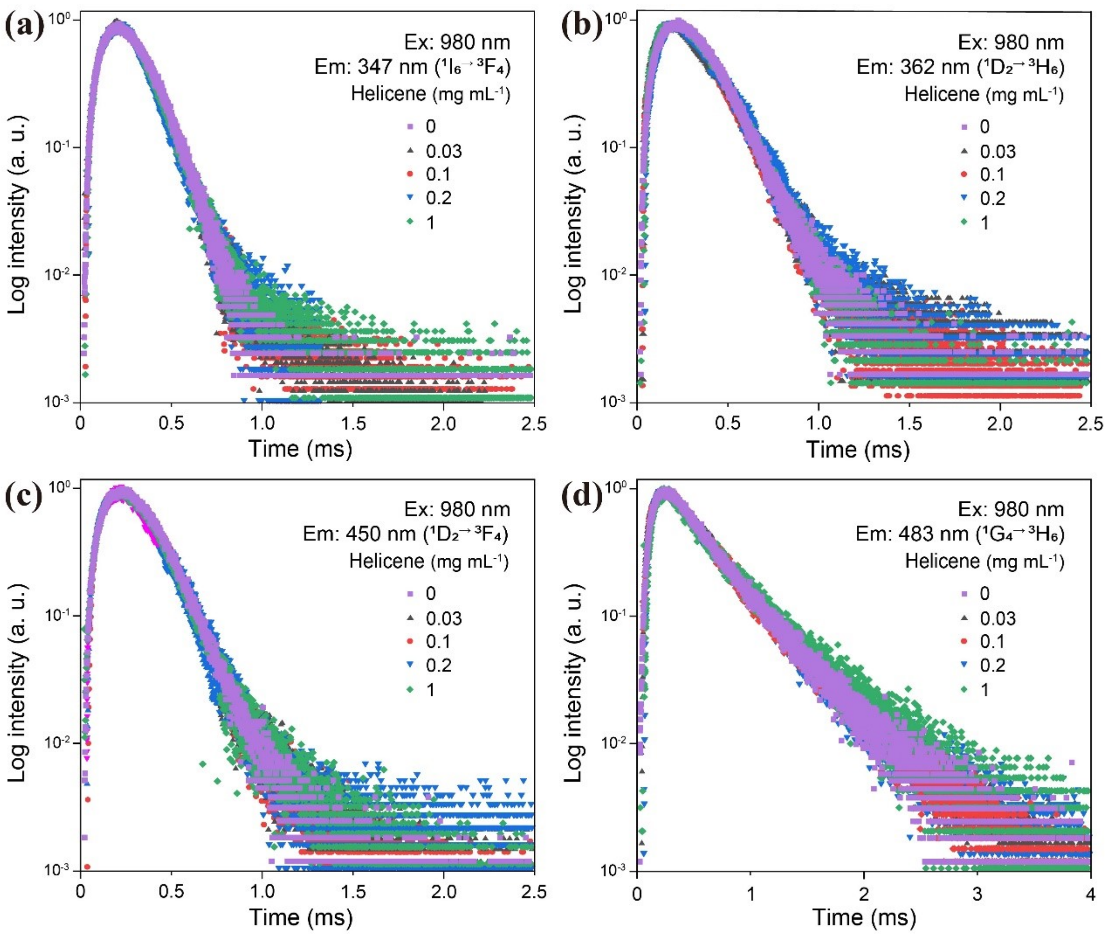

Figure S17. Helicene concentration-dependent UCL decays from a) $^1I_6$, b-c) $^1D_2$, and d) $^1G_4$ of Tm^{3+} by monitoring the Tm^{3+} emissions at 347, 362, 450, and 483 nm, respectively, in the NP-sensitized helicene with NPs concentration of 40 mg mL^{-1} under 980 nm excitation. The UCL decay profiles are nearly identical for each level of Tm^{3+} and show no dependent on the concentration or absorption of helicene, thus providing evidences for radiative energy transfer from the NPs to helicene.

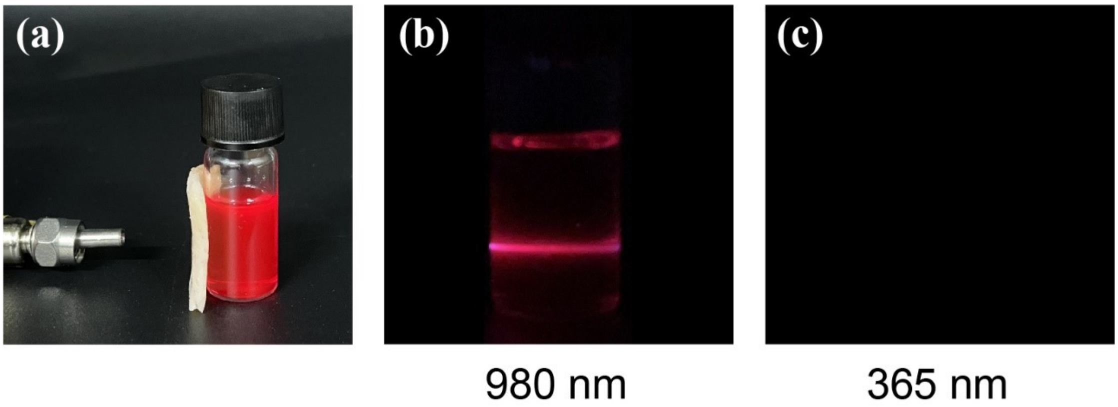

Figure S18. Schematic diagram of the penetration experiment using pig skin tissue a-b) under 980 nm excitation and c) under 365 nm excitation. Under 980 nm laser excitation, NPs-helicene emitted red light, whereas 365 nm excitation failed to penetrate the tissue effectively.

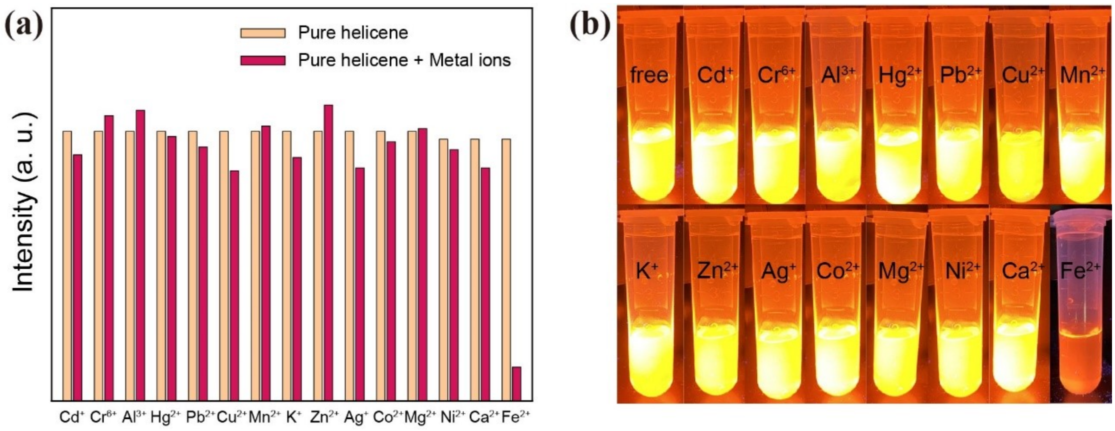

Figure S19. a) Selectivity graphs evaluated in helicene for Fe^{2+} and other metal ions (λ_{ex} = 365 nm). b) The luminescence photos after adding Fe^{2+} and other metal ions to helicene (λ_{ex} = 365 nm). The comparison of fluorescence intensity confirms that the fluorescence quenching effect in helicene occurs only upon contact with Fe^{2+}, while other common metal ions do not impact the fluorescence.

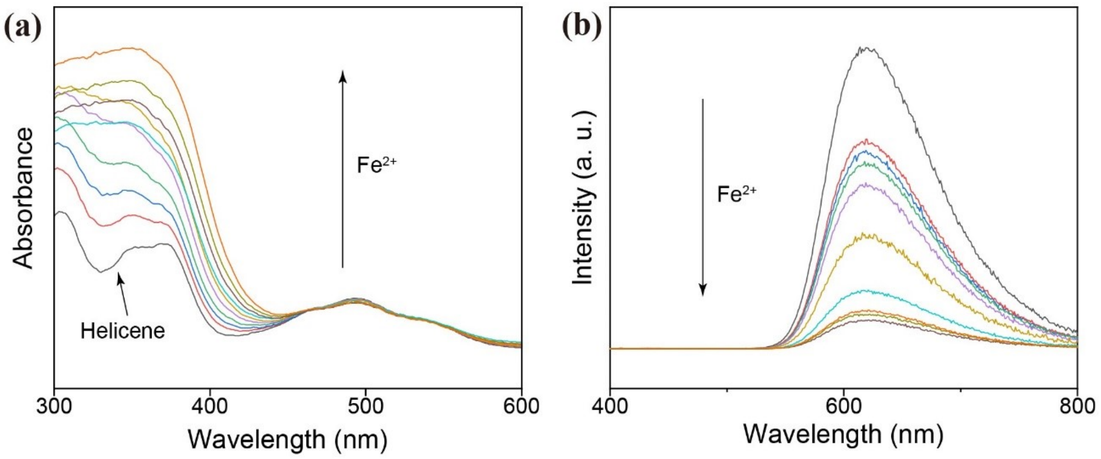

Figure S20. a) UV–vis absorption spectra and b) emission spectra for 1 mg helicene in CH_2Cl_2 with the addition of different concentration of Fe^{2+} (λ_{ex} = 365 nm). With the gradual addition of Fe^{2+}, the absorbance of helicene gradually increased, indicating that the molecular structure of helicene undergoes a change upon encountering Fe^{2+}. With the addition of Fe^{2+}, the fluorescence intensity gradually decreased.

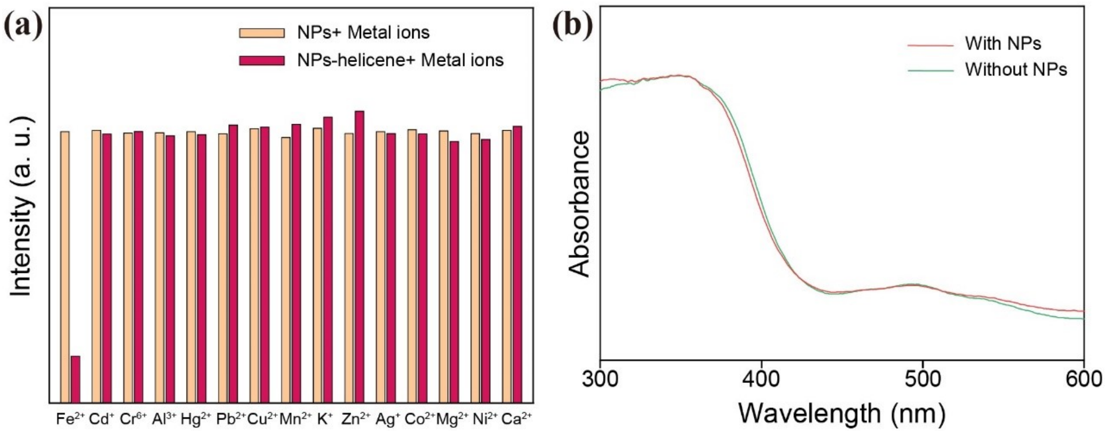

Figure S21. a) Selectivity graphs evaluated in NPs (yellow) and NPs-helicene (red) for Fe^{2+} and other metal ions (λ_{ex} = 980 nm). b) UV–vis absorption spectra for helicene and NPs-helicene. The selectivity graphs clearly indicate that the NPs do not possess Fe^{2+} recognition capability, and their incorporation does not interfere with the Fe^{2+} detection activity of helicene. Furthermore, the UV-vis absorption spectra of helicene and NPs-helicene confirm that the presence of NPs has no impact on the visible absorption properties of helicene, demonstrating that the photophysical characteristics of helicene remain unchanged upon integration with NPs.

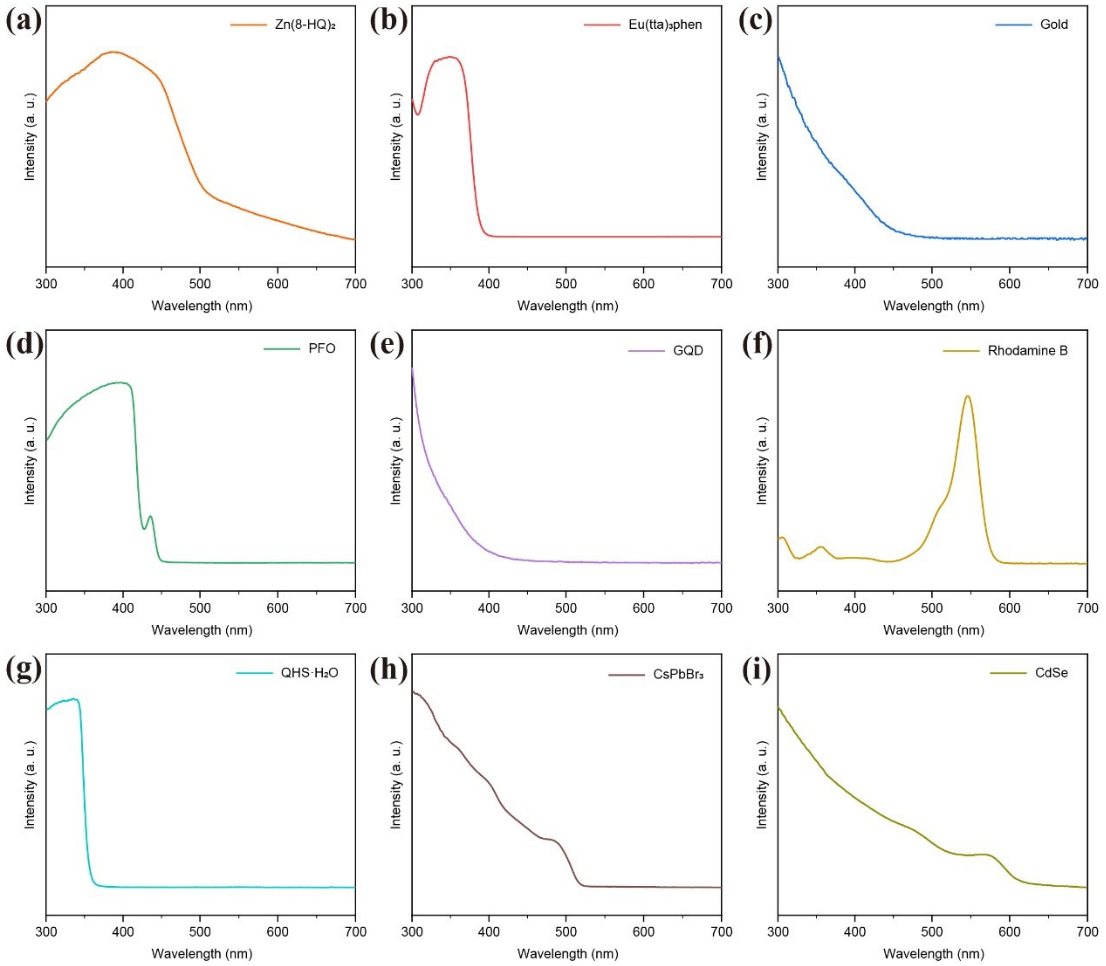

Figure S22. Absorption spectra of a) Zn(8-HQ)$_2$, b) Eu(tta)$_3$phen, c) gold clusters, d) Poly(9,9-dioctylfluorene-2,7-diyl) (PFO), e) Graphene quantum dots (GQDs), f) Rhodamine B, g) Quinine hemisulfate monohydrate (QHS·H_2O), h) $CsPbBr_3$ quantum dots (QDs), i) CdSe QDs. These materials exhibit excellent absorption characteristics, ranging from the UV region to the red region, which align with the emission peaks of lanthanide-doped NPs.

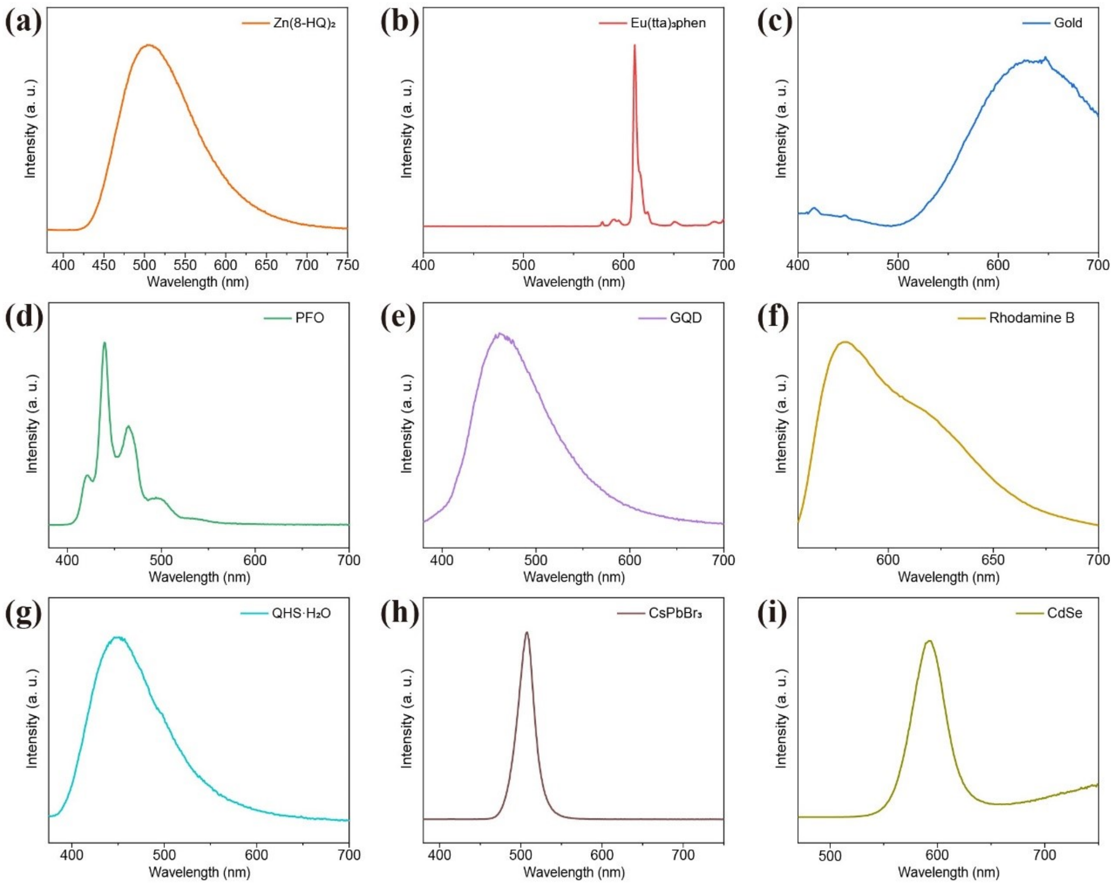

Figure S23. PL emission spectra of a) Zn(8-HQ)$_2$, b) Eu(tta)$_3$phen, c) Gold clusters, d) Poly(9,9-dioctylfluorene-2,7-diyl) (PFO), e) Graphene quantum dots (GQDs), f) Rhodamine B, g) Quinine hemisulfate monohydrate (QHS·H$_2$O), h) CsPbBr$_3$ quantum dots (QDs), i) CdSe QDs, under 365 nm excitation.

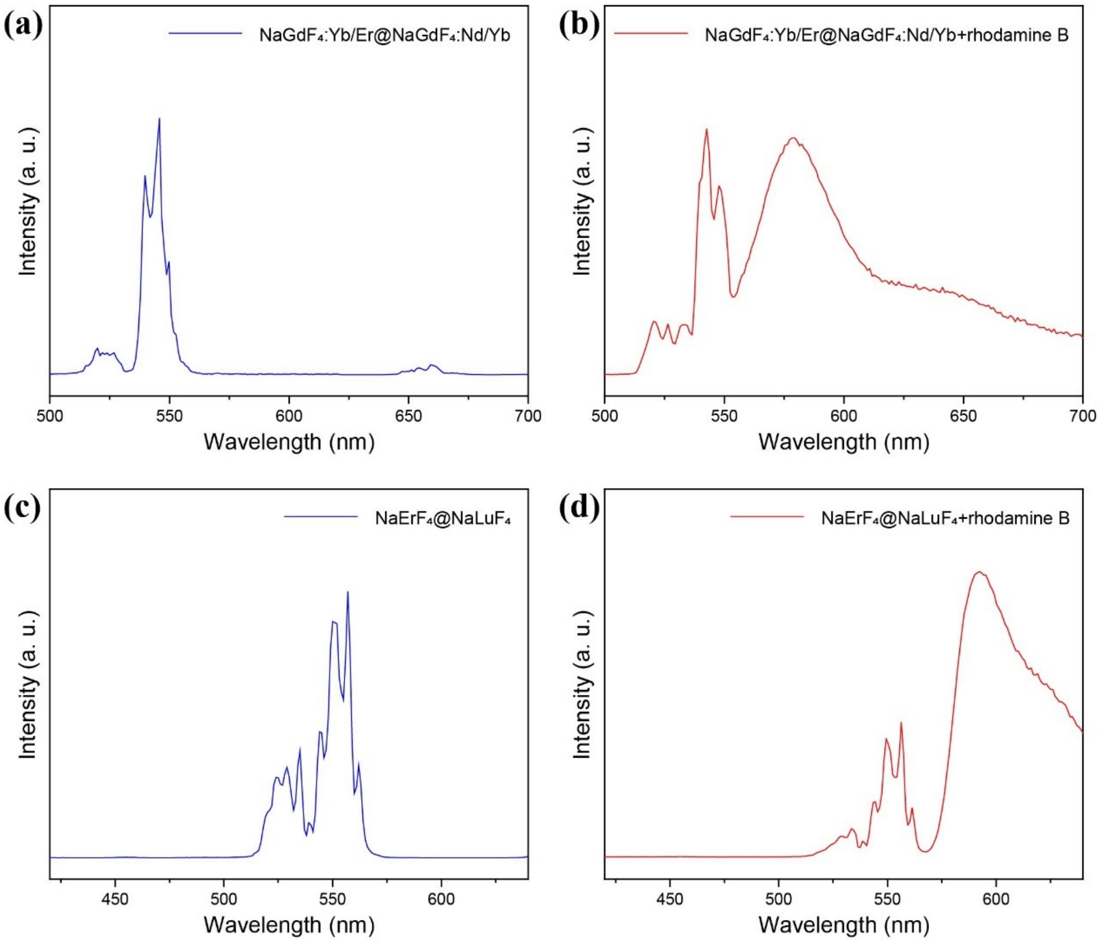

Figure S24. UCL spectra of a) $NaGdF_4$:20%Yb/2%Er@$NaGdF_4$:10%Nd/10%Yb NPs and b) $NaGdF_4$:20%Yb/2%Er@$NaGdF_4$:10%Nd/10%Yb NPs-rhodamine B, under 808 nm excitation. UCL spectra of c) $NaErF_4$@$NaLuF_4$ NPs and d) $NaErF_4$@$NaLuF_4$ NPs-rhodamine B, under 1532 nm excitation.

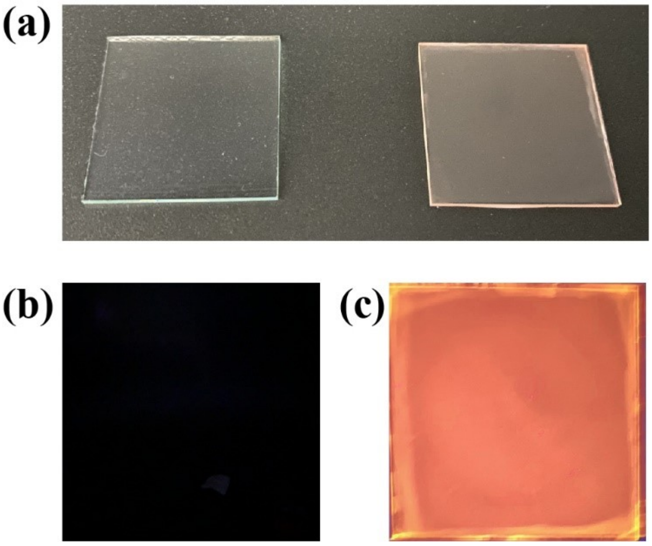

Figure S25. a) Schematic illustration of square glass substrates with blank control plate (left) and NPs-rhodamine B blend films (right). b) Luminescent images of the blank plate and c) blend films on the square glass substrates under 980 nm excitation. The NPs-rhodamine B solution was drop-cast onto the square glass substrates to form blend films. Under 980 nm excitation, the blank plate showed no response, the NPs-rhodamine B blend films emitted bright red light.

References

[1] H.-X. Mai, Y.-W. Zhang, R. Si, Z.-G. Yan, L.-D. Sun, L.-P. You, and C.-H. Yan, *J. Am. Chem. Soc.* 2006, **128**, 6426-6436

[2] W. W. Yang, Z. H. Ren, J. Feng, Z. B. Lv, X. W. Cheng, J. M. Zhang, D. L. Du, C. Y. Chi and J. J. Shen, *Angew. Chem. Int. Ed.*, 2024, **63**, e202412681.
